# Systematic benchmarking and optimal strategy selection of cross-species integration methods

**DOI:** 10.1093/bib/bbag490

**Published:** 2026-09-10

**Authors:** Ruolin Wang, Junjuan Zheng, Chuning Mao, Ya-Ping Zhang, Zhaoli Ding, Guo-Dong Wang

**Affiliations:** State Key Laboratory of Genetic Evolution and Animal Models, Kunming Institute of Zoology, Chinese Academy of Sciences, 17 Longxin Road, Kunming, Yunnan, 650201, China; Kunming College of Life Science, University of Chinese Academy of Sciences, 19 Qingsong Road, Kunming, Yunnan, 650201, China; State Key Laboratory of Genetic Evolution and Animal Models, Kunming Institute of Zoology, Chinese Academy of Sciences, 17 Longxin Road, Kunming, Yunnan, 650201, China; Kunming College of Life Science, University of Chinese Academy of Sciences, 19 Qingsong Road, Kunming, Yunnan, 650201, China; State Key Laboratory of Genetic Evolution and Animal Models, Kunming Institute of Zoology, Chinese Academy of Sciences, 17 Longxin Road, Kunming, Yunnan, 650201, China; Kunming College of Life Science, University of Chinese Academy of Sciences, 19 Qingsong Road, Kunming, Yunnan, 650201, China; State Key Laboratory of Genetic Evolution and Animal Models, Kunming Institute of Zoology, Chinese Academy of Sciences, 17 Longxin Road, Kunming, Yunnan, 650201, China; Kunming College of Life Science, University of Chinese Academy of Sciences, 19 Qingsong Road, Kunming, Yunnan, 650201, China; Bio-X Center for Interdisciplinary Innovation, Yunnan University, 2 Cuihu North Road, Kunming, Yunnan 650091, China; State Key Laboratory of Genetic Evolution and Animal Models, Kunming Institute of Zoology, Chinese Academy of Sciences, 17 Longxin Road, Kunming, Yunnan, 650201, China; Kunming College of Life Science, University of Chinese Academy of Sciences, 19 Qingsong Road, Kunming, Yunnan, 650201, China; State Key Laboratory of Genetic Evolution and Animal Models, Kunming Institute of Zoology, Chinese Academy of Sciences, 17 Longxin Road, Kunming, Yunnan, 650201, China; Kunming College of Life Science, University of Chinese Academy of Sciences, 19 Qingsong Road, Kunming, Yunnan, 650201, China

**Keywords:** single-cell transcriptome, cross-species comparison, integrated evaluation

## Abstract

Single-cell RNA sequencing provides an unprecedented resolution for cellular heterogeneity and gene regulation, fostering cross-species comparative analyses with increasing interspecies data. However, integrating single-cell transcriptomic data faces challenges, including gene selection, evolutionary distance, and batch effects, with varying method performances. We utilized single-cell transcriptomic data from hippocampal tissues of seven mammals (e.g. mouse, human), evaluating 13 mainstream integration methods across 27 tasks with 11 metrics. To compare the performance of different methods, we developed a machine learning-based scoring model that assesses the contribution of each metric in a data-driven manner, thereby addressing the oversimplified assumptions of traditional manual weighting approaches. Our findings show that selecting highly variable one-to-one orthologous genes best balances species differences and commonalities. Most methods integrated closely related species, whereas scVI, a probabilistic model with distributions specified by deep neural networks, and its semi‑supervised extension scANVI, as well as the Seurat v5 method, which uses reciprocal principal component analysis (RPCAv5), effectively mapped distantly related species. Increased species numbers reduce gene overlap and heighten heterogeneity, increasing integration difficulty. The scANVI best maintained quality by balancing the biological signals and batch effect removal. Furthermore, we established an evaluation website to guide researchers in selecting the optimal integration methods for cross-species single-cell transcriptomic data analysis. Collectively, our findings provide a systematic, evidence-based framework that can assist researchers in rapidly selecting appropriate integration methods for cross-species single-cell transcriptomic studies.

## Graphical Abstract

**Graphical Abstract:**
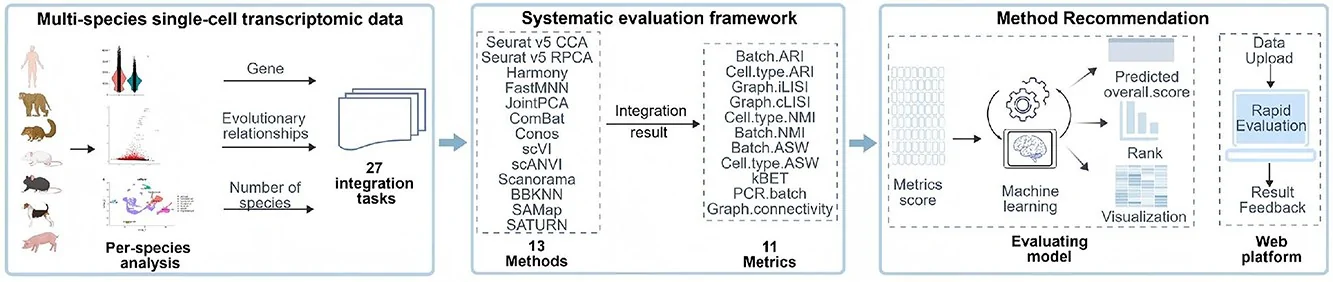

## Introduction

The rapid development of single-cell RNA sequencing (scRNA-seq) technology has significantly enhanced our understanding of complex biological systems and enabled cellular-level analysis of genetic variations and evolutionary mechanisms across species [1–4]. In particular, conducting cross-species comparative analyses of cell types at the tissue or organ level has revealed cell type conservation and diversity during species evolution, offering new insights into evolutionary mechanisms [5–7]. With advancing technology and decreasing sequencing costs, increasing single-cell transcriptomic data from diverse species have laid a solid foundation for systematic cross-species cellular comparison and evolutionary studies [8, 9].

However, achieving high-quality cross-species single-cell transcriptomic data integration faces multiple challenges [10]. First, interspecies genome structure and annotation differences limit high-quality one-to-one orthologous (O2O) gene set generation for alignment [11]; strict O2O sets reduce noise, but cause substantial biological gene loss [12, 13]. This issue becomes even more pronounced as the number of species to be integrated increases [14]. Second, data from different batches and workflows affect the results, with cell type differences masked by species or technical variations [15]. Additionally, uneven data volume may overlook rare cell types, weakening biological interpretability [16–18]. Furthermore, large evolutionary distances pose challenges for species-specific cell type annotation and matching [19, 20].

Recently, several studies have systematically compared cross-species single-cell transcriptomic integration methods. Song *et al*. [21] and Zhong *et al*. [22] designed multiple integration tasks to systematically assess the performance of various mainstream integration methods to preserve biological features and eliminate batch effects. These studies revealed differential performances among methods in capturing biological differences across species, offering valuable references for selecting appropriate cross-species single-cell transcriptomic data integration approaches. However, we still face limitations in systematicity and practicality: newly developed or updated methods are not fully covered, failing to reflect the latest integration advancements; existing evaluation metrics vary in applicability and rely on simple empirical formulas, reducing the reference value of the evaluation results. Therefore, designing more realistic cross-species integration task frameworks, enhancing the comprehensiveness of performance comparisons, updating integration methods to systematically clarify the applicability and advantages of different approaches, and providing practical method selection guidance for researchers remains an important open question in cross-species benchmarking.

**Figure 1:**
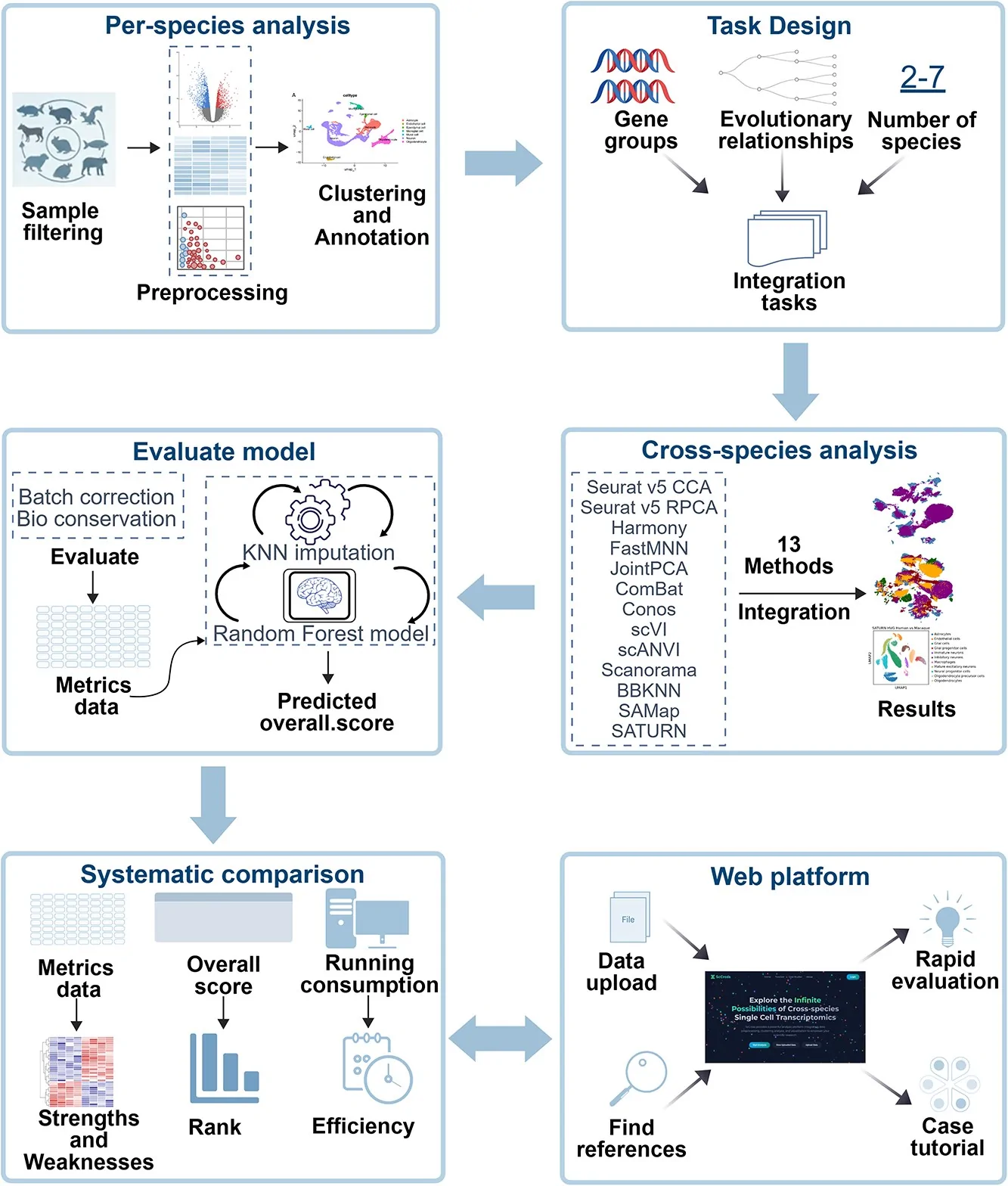
Schematic of the benchmarking workflow, created with BioGDP.com [31]; first, we processed the data involved in the study, including quality control and normalization; then, cross-species analysis is conducted based on different evolutionary relationships, species numbers, and gene groups, resulting in diverse clustering outcomes; the integrated results are evaluated, focusing on batch correction and biological conservation, with scoring and ranking done through a model; finally, a web platform is provided to support researchers in exploring the potential of cross-species single-cell transcriptomics.

Here, we address a fundamental need for cross-species single-cell transcriptomics: how to systematically benchmark and integrate hippocampal scRNA-seq data across evolution, while maintaining both technical consistency and biological fidelity. We systematically designed 27 cross-species integration tasks based on three key dimensions: gene selection strategies, evolutionary relationships, and number of species. We chose the hippocampus as the research object because of its high conservation and strong comparability [23]. We selected seven species spanning major evolutionary branches from primates to rodents [24–29], including *Mus musculus* (Mouse), *Rattus norvegicus* (Rat), *Tupaia belangeri* (Tree shrew), *Homo sapiens* (Human), *Macaca mulatta* (Rhesus macaque), *Canis lupus familiaris* (Dog), and *Sus scrofa* (Pig). For method benchmarking, we evaluated 13 representative integration methods: the Seurat v5 method, which uses canonical correlation analysis (CCA) or reciprocal principal component analysis (RPCA); joint principal component analysis (JointPCA); an empirical Bayes‑based batch‑effect correction method (ComBat); mutual nearest neighbors (fastMNN) and its variations (Scanorama); the k‑means algorithm (Harmony); scVI, a probabilistic model with distributions specified by deep neural networks, and its semi‑supervised extension scANVI; a graph‑based method for integrating multiple single‑cell datasets (Conos); batch‑balanced k‑nearest neighbors (BBKNN); and gene‑sequence‑alignment‑based integration methods (SAMap and SATURN). To enable a scientific objective comparison across diverse scenarios, we built a machine-learning-based scoring model over an evaluation system that separates batch correction and biological conservation [30]. Finally, to support practical adoption, we established a one-stop cross-species single-cell transcriptome integration evaluation platform that aims to simplify complex bioinformatics workflows, enabling researchers without programming backgrounds to conduct professional single-cell data studies (Fig. 1).

## Materials and methods

### Datasets and preprocessing

We conducted a comprehensive benchmarking of mainstream cross-species integration methods using seven published single-cell transcriptomic datasets (Supplementary Table S1). To ensure data consistency during the preprocessing stage, all datasets were processed using Seurat v5.3.0 [31] with unified settings. Default parameters were used, including min.cells = 3, min.features = 200, filtering thresholds of nFeature_RNA >200 and nFeature_RNA <2500, normalization using scale.factor = 10 000, and dimensionality reduction with npcs = 50. We compiled all previously published cell type annotations across the datasets in this study as a reference baseline for annotation and performed manual curation. In particular, we ensured that the same cell type identifier consistently corresponded to homologous cell types, thereby improving the accuracy of the results. During the integration process, to ensure fair comparability and reproducibility across different methods and tasks, we set the hyperparameters of the algorithms to their default values or fixed configurations specified in official tutorials and recommended workflows [21, 22]. We obtained lists of O2O genes from the Ensembl database [32] and selected 2000 highly variable genes (HVGs) from them. Additionally, to accommodate methods dependent on sequence information, we supplemented the necessary protein and nucleic acid sequence similarity data for SAMap and completed protein language model embedding for SATURN (Supplementary Table S2).

### Cross-species data integration methods

#### Seurat v5 (CCA) and Seurat v5 (RPCA)

This study used Seurat v5.3.0 to implement the CCA v5 method (Supplementary Table S3), which identifies “anchor pairs” via CCA-constructed shared subspace, integrates raw data, and projects them to a common hyperplane [33]. Similarly, RPCA v5 (Supplementary Table S3) first performs individual principal component analysis (PCA) to identify “anchor pairs” for the efficient integration of large/heterogeneous datasets [31]. Two strategies (O2O and 2000 HVGs) were used; postintegration, results were standardized and transformed to PCA space, and the top 30 principal components (PCs) were extracted.

#### Harmony

Harmony [34] used an iterative clustering algorithm to eliminate the batch effects (Supplementary Table S3). It groups cells to maximize cluster diversity, calculates cluster centroids and correction factors, iteratively adjusts cell parameters until convergence, and generates corrected embedded vectors. The Harmony run in R used two strategies (O2O and 2000 HVGs) after data normalization and scaling: max clusters = 50, max iterations = 100 (default otherwise), with corrected vectors for subsequent evaluation.

#### FastMNN

FastMNN [35], based on MNN, mitigated batch effects via MNN pairs identified in the PCA space (Supplementary Table S3). After data standardization and normalization, two strategies (O2O and 2000 HVGs) were used. Using the default parameters of FastMNN, the generated embedding vectors were extracted for subsequent evaluation and analysis.

#### JointPCA

JointPCA [31] learns a shared PCA space across datasets to build neighbor graphs and correct batch effects, thereby generating integrated representations (Supplementary Table S3). After data standardization and normalization, two strategies (O2O and 2000 HVGs) were used for PCA, enabling cross-species batch difference correction in a unified low-dimensional space.

#### ComBat

This study employed ComBat [36] in the sva package for cross-species single-cell batch correction (Supplementary Table S3). As a classical parametric method, it estimated the mean and variance by using a Bayesian framework to correct batch variations. After data standardization and normalization, expression matrices were built using O2O and 2000 HVGs, and the species origin was used as a covariate for batch correction.

#### Conos

Conos [37] used graph theory and network clustering for robust cross-species single-cell integration (Supplementary Table S3), which was suitable for complex and heterogeneous systems. It constructed inter-sample cell-similarity networks for anchor-free many-to-many integration. After data standardization and normalization, O2O and 2000 HVGs were used to build adjacency networks, integrate them into a unified graph, and achieve network alignment while preserving heterogeneity and shared features.

#### scVI and scANVI

This study applied the scVI [38], scANVI [39], and neural network models based on hierarchical Bayesian frameworks for cross-species data integration (Supplementary Table S3). These models capture dataset-specific random effects via a negative binomial distribution to mitigate batch effects and cell type heterogeneity. After data standardization and normalization, analyses were performed using O2O and 2000 HVGs. scVI embeds data into a 50D latent space with default parameters. It is noteworthy that, although scANVI can be initialized from a pretrained scVI model, we built the scANVI model from scratch using the input AnnData object to ensure that the operation of scANVI remained independent of scVI in benchmarking tests. Furthermore, as a semi-supervised model, scANVI employs label constraints on the labeled subset with labels_key = “celltype” during training, with all parameters set to the default values.

#### Scanorama

Scanorama [40] was designed for single-cell transcriptome integration by identifying mutual nearest neighbors across datasets (Supplementary Table S3). After standardization and normalization, Scanorama was applied using O2O and 2000 HVGs. The batch size was set to 30, k-nearest neighbor (kNN)to 10, and the return dimension was set to be true with other default parameters.

#### B‌BKNN

BBKNN [41] uses k-nearest neighbor mapping to capture cellular similarities (Supplementary Table S3). We used the R wrapper for the analysis. After data normalization, O2O and 2000 HVGs from O2O were used; they calculated intra-batch cell distances, identified k-nearest neighbors, merged inter-batch maps, and aligned cells via shared nearest neighbors.

#### SAMap

SAMap [42] integrates cross-species single-cell datasets by combining protein sequence similarity and gene expression correlations (Supplementary Table S3). It identifies cross-species MNN considering both factors to find meaningful intercellular correspondences, and then uses these MNN to transform different species’ feature spaces into a unified space.

#### Saturn

SATURN [43] is a deep learning method for cross-species single-cell transcriptome integration (Supplementary Table S3). It combines scRNA expression and protein embedding vectors from a protein language model using a pretrained conditional autoencoder to map data to a low-dimensional space.

### Dimension reduction and visualization

In this study, for dimensionality reduction and visualization, we performed PCA using RunPCA (npcs = 50) and computed the uniform manifold approximation and projection (UMAP) in the integrated space with RunUMAP (dims = 1:50). Next, we constructed a neighbor graph using FindNeighbors (dims = 1:30) and identified the cell clusters using FindClusters. Finally, cell distributions in the low-dimensional space were visualized using DimPlot based on the UMAP embedding, with analyses performed separately according to group.by = “species” and group.by = “celltype.” SAMap and SATURN output a corrected neighborhood graph, so we only recalculated the UMAP using the same parameters for visualization.

### Evaluated metrics

This study established an evaluation system based on 2D: batch-effect removal and biological preservation. In the assessment of batch effects, metrics such as the Local Inverse Simpson’s Index (LISI) batch score, Normalized Mutual Information (NMI) batch effect, Adjusted Rand Index (ARI) batch effect, average silhouette width (ASW) batch effect [44], kNN batch effect test (kBET) [45], and principal component regression (PCR) [30], as well as graph connectivity [30], were employed to quantitatively describe the residual degree of batch effects in the integrated data through different computational methods. The evaluation metrics for biological retention include LISI, NMI, ARI, and ASW cell type, with the aim of detecting the retention degree of cell type information and the integrity of biological signals after integration.

#### Local Inverse Simpson’s Index

The LISI [34] score metric calculates scores for both batch graph Local Inverse Simpson’s Index (iLISI) and cell type (cLISI), reflecting the degree of batch mixing and the preservation of cell type information, respectively. The LISI score ranges from 1 to the upper limit of the number of unique cell types or unique batches in the dataset. A higher iLISI score indicates a greater number of batches, indicating better batch mixing. A higher cLISI score suggests a greater number of cell types in the group, indicating poorer preservation of cell type information. In calculating LISI scores, we adopted the normalization procedure described by Tran *et al*. [44]: for the method of outputting pseudo-count matrices, k-nearest neighbors were calculated using the first 30 principal components; for the method of directly generating embedded vectors, k-nearest neighbors were calculated using the embedded vectors. To normalize LISI scores, we applied the following formulas to cLISI and iLISI to standardize them to the 0–1 range.


(1) \begin{equation*} \mathrm{iLISInormalized}=\frac{\mathrm{iLISI}-1}{\mathrm{unique}\_\mathrm{batch}\_\mathrm{amount}-1} \end{equation*}

(2) \begin{equation*} \mathrm{cLISInormalized}=\frac{\mathrm{unique}\_\mathrm{celltype}\_\mathrm{amount}-\mathrm{cLISI}}{\mathrm{unique}\_\mathrm{celltype}\_\mathrm{amount}-1} \end{equation*}

#### NMI

NMI quantifies clustering result similarity (0–1), with 1 indicating complete consistency and 0 indicating randomness [30]. We used the single-cell integration benchmark (scIB) workflow to compare cell type, batch (species) labels, and Louvain clustering. A higher cell type NMI indicates better cell type preservation; batch NMI (1 – NMI_raw_) is higher with better batch mixing.


(3) \begin{equation*} \mathrm{Batch}\ \mathrm{NMI}=1-\mathrm{NMIraw} \end{equation*}

#### ARI

ARI [46] measures the similarity between the assigned and true labels to reflect integration accuracy, calculated via scikit-learn’s adjusted_rand_score (20 repetitions for robustness). ARIc (per cell type average) indicates cell type matching, while ARIb (all cell types average) is adjusted to ensure that higher scores mean better batch effect elimination.


(4) \begin{equation*} \mathrm{Batch}\ \mathrm{ARI}=1-\mathrm{ARIb} \end{equation*}

#### Average silhouette width

The ASW metric evaluates clustering quality by assessing both intra-cluster cohesion and inter-cluster separation. For each cell, the contour width method first calculates the minimum average distance between the cell and other cells outside its cluster, then subtracts the average distance within the same cluster, and finally divides the result by the larger of the two values. The ASW metric is derived by averaging the contour widths of all clustered cells, with values ranging from –1 to 1. Higher values indicate better clustering quality, which helps assess the retention of cell type information and the effectiveness of batch mixing. Referencing Tran *et al*. [44], we adopted a unified computational process to evaluate ASW scores from both batch and cell type dimensions. By repeating this process 20 times, we obtained 20 sets of ASW scores under both batch-mixing and cell type conservation conditions. All ASW scores were subsequently normalized using the following formula to convert them into values within the range of 0–1: 


(5) \begin{equation*} \mathrm{ASWnormalized}=\frac{\mathrm{ASW}+1}{2} \end{equation*}

For batch mixing, we applied the following equation to scale the normalized batch ASW scores, with higher scores indicating better interspecies mixing: 


(6) \begin{equation*} Batch\ ASW=1- ASWnormalized \end{equation*}

#### kNN batch effect test

The kBET [45] is a metric designed to quantify the degree of batch mixing in integrated datasets. This method assesses the extent of sample mixing from different batches within repeated random subsampling units. A higher kBET score indicates greater batch mixing, suggesting successful elimination of batch effects and enhanced integration of data across batches. Conversely, a lower score reflects reduced batch mixing, implying a better preservation of batch-specific signals and diminished undesirable batch effects in the integrated dataset. The extended kBET procedure proposed in the scIB workflow [30] was employed to calculate the score.

#### Principal component regression

PCR [30] is an indicator used to quantify the elimination of batch effects using integration methods. This metric is based on the assumption that batch variables are correlated with principal components derived from the integrated dataset. It is applicable only to non-plot-type output methods. In calculating the score, we follow the scIB workflow [30]. First, we perform a default analysis of 50 principal components on the pseudo-count output and embedded output to identify the principal components that capture the primary sources of data variation. Then, we establish a relationship model between batch variables and each principal component through linear regression. Finally, we derive the total score by summing the variance contribution values of batch variables across all principal components. By evaluating the correlation between batch variables and principal components, the PCR score reveals the effectiveness of integration methods in eliminating batch effects. A higher score indicates a greater reduction in batch-related variation, suggesting better elimination of batch effects in the integrated dataset.

### Graph connectivity

Graph connectivity evaluates connectivity of same-label cells in kNN graphs. Following scIB [30], we build a kNN graph (*k* = 50) from pseudo-counts or embeddings, subsample the largest same-label component, and average the ratios of component nodes to total label nodes. Scores (0–1) are higher with stronger same-label cell connectivity.

### Ranking and metric aggregation

Systematic evaluation of integration outcomes is critical for cross-species single-cell transcriptome integration studies. Current methods assess batch effect elimination and biological conservation with total scores obtained via empirical weighting. Researchers prioritize biological conservation, using 0.6 (biological conservation) and 0.4 (batch effect elimination) weights [22], but this system lacks a theoretical basis and extensive validation.


(7) \begin{align*} \mathrm{Soverall}=\,&\frac{1}{\mathrm{n}}\sum \limits_{i=1}^n(0.4\times \mathrm{Sbatch}-\mathrm{correction},\mathrm{i}+0.6\nonumber\\&\times \mathrm{Sbioconservation},\mathrm{i}) \end{align*}

Most evaluation metrics are designed for traditional integration methods. Emerging methods (e.g. SAMap and SATURN) that follow different methodologies often make metrics uncomputable (NA). Although preprocessing (e.g. max-min scaling) ensures total score reliability, NA values cause incomparability; arbitrarily excluding computable metrics would further reduce assessment comprehensiveness.

To mitigate these issues, this study develops a more objective assessment framework. To address the impact of missing values, we use KNN interpolation [47] to fill them, which identifies similar samples and estimates missing values from the K most similar ones, preserving the data structure and feature relationships.


(8) \begin{equation*} \mathrm{xij}=\frac{\sum_{\mathrm{m}\in \mathrm{Ni}}\mathrm{wim}\mathrm{xmj}}{\sum_{\mathrm{m}}\mathrm{wim}},\quad \mathrm{wim}=\frac{1}{\mathrm{d}\left(\mathrm{xi},\mathrm{xm}\right)} \end{equation*}

In this formula, *X_ij_* is the *j*th feature value of the *i*th sample, *N_i_* is the k-nearest neighbors of sample *i*, *X_mj_* is the *j*th feature’s known value of neighbor *m*, and *W_im_* [inverse of distance *d*(*X_i_*,*X_m_*)] is the weight of sample *i* to *m*, with closer distance meaning higher weight.

In addition, to eliminate differences in the dimensions of the different metrics, we apply MinMaxScaler to normalize all evaluation metrics.


(9) \begin{equation*} \mathrm{Xnormalized}=\frac{\mathrm{X}-\mathrm{Xmin}}{\mathrm{X}\mathrm{max}-\mathrm{Xmin}} \end{equation*}

*X* is the original indicator value and *X*_min_ and *X*_max_ are the minimum and maximum values, respectively. Normalization confines values to [0,1], ensuring equal weighting for subsequent modeling.

We adopted the Random Forest Regressor [48] for the scoring system. It is an ensemble method with strong noise resistance and good generalization performance, making it suitable for bioinformatic datasets with limited samples and high dimensionality. It builds multiple decision trees and averages their predictions. The regression principle is as follows:


(10) \begin{equation*} y\hat{\mkern6mu} =\frac{1}{\mathrm{T}}{\sum}_{\mathrm{t}=1}^{\mathrm{T}}\mathrm{ht}\left(\mathrm{x}\right) \end{equation*}

Here, $\hat{y}$ denotes the final prediction, *T* represents the total number of decision trees in the Random Forest, and *h_t_*(*x*) indicates the prediction value assigned by the *t*th decision tree to sample *x*.

During the model construction, we used a grid search to find the model with the lowest empirical risk. Cross-validation (CV) [49] evaluated the generalization of each parameter combination to determine the optimal settings (n_estimators, max_depth, min_samples_split, min_samples_leaf, max_features = “sqrt”). We performed 10 repeated K-fold CV [50] to reduce the split variability and obtain robust performance estimates.

### Website design and visualization

The Website design and visualization method of the Single-cell Cross-species Analysis Platform is based on Flask [51] for backend and RESTful application programming interface (API). We employed SQLite [52], an in‑process, serverless, zero‑configuration, transactional Structured Query Language (SQL) database engine, for data persistence, and utilized Flask‑CORS, a Flask extension implementing Cross‑Origin Resource Sharing (CORS), to handle cross‑origin requests. We used Scanpy [53] for data processing, parameterized queries to prevent SQL [54] injection, secure configuration management [55], flexible deployment with error handling [56], Python packages including Pandas [57] and SciPy [58], and integrating Layui (https://layui.dev/) for frontend static assets.

## Results

### Multidimensional cross-species single-cell transcriptome integration framework

To benchmark the cross-species integration methods, we obtained hippocampal single-cell transcriptomic data from seven mammalian species (Table 1). The mammalian hippocampus, as an ideal model tissue with a rich evolutionary representation, provides optimal conditions for evaluation.

**Table 1:** Raw data sources and information.

**Species**	**Sources**	**Dataset**	**Technology**	**Number of cells**
*Homo sapiens*	GEO	GSE186538	10× chromium	23 567
*Macaca mulatta*	GEO	GSE186538	10× chromium	36 066
*Rattus norvegicus*	GEO	GSE232936	10× chromium	7755
*Mus musculus*	GEO	GSE158450	10× chromium	16 063
*Sus scrofa*	GEO	GSE186538	10× chromium	27 295
*Canis lupus familiaris*	CNCB	PRJCA004294	SPLiT-seq	105 057
*Tupaia belangeri*	GEO	GSE229035	10× chromium	29 817

The Table 1 is located in the first paragraph of the multidimensional cross-species single-cell transcriptome integration framework, in the results section.

In the design of integration tasks, we constructed 27 systematic and representative integration tasks (Supplementary Table S4). First, to systematically analyze the impact of evolutionary relationships on cross-species integration, we selected four species to establish three different levels of evolutionary relationships: closely related (human and rhesus macaque), moderately related (human and mouse), and distantly related (mouse and pig). Then, to explore the effect of species number on integration, we divided the integration tasks into different scales, designing multiscale tasks ranging from two species to seven species combinations. Regarding gene selection strategies, considering the requirements of different algorithms and the complexity of cross-species co-expression characteristics, we applied O2O, HVGs, and all genes (ALL) to different integration methods (Seurat v5 CCA, Seurat v5 RPCA, Harmony, FastMNN, JointPCA, ComBat, Conos, scVI, scANVI, Scanorama, and BBKNN used O2O and HVGs; SAMap used ALL genes; and SATURN used HVGs) to explore how to select appropriate gene strategies for better integration outcomes.

**Figure 2:**
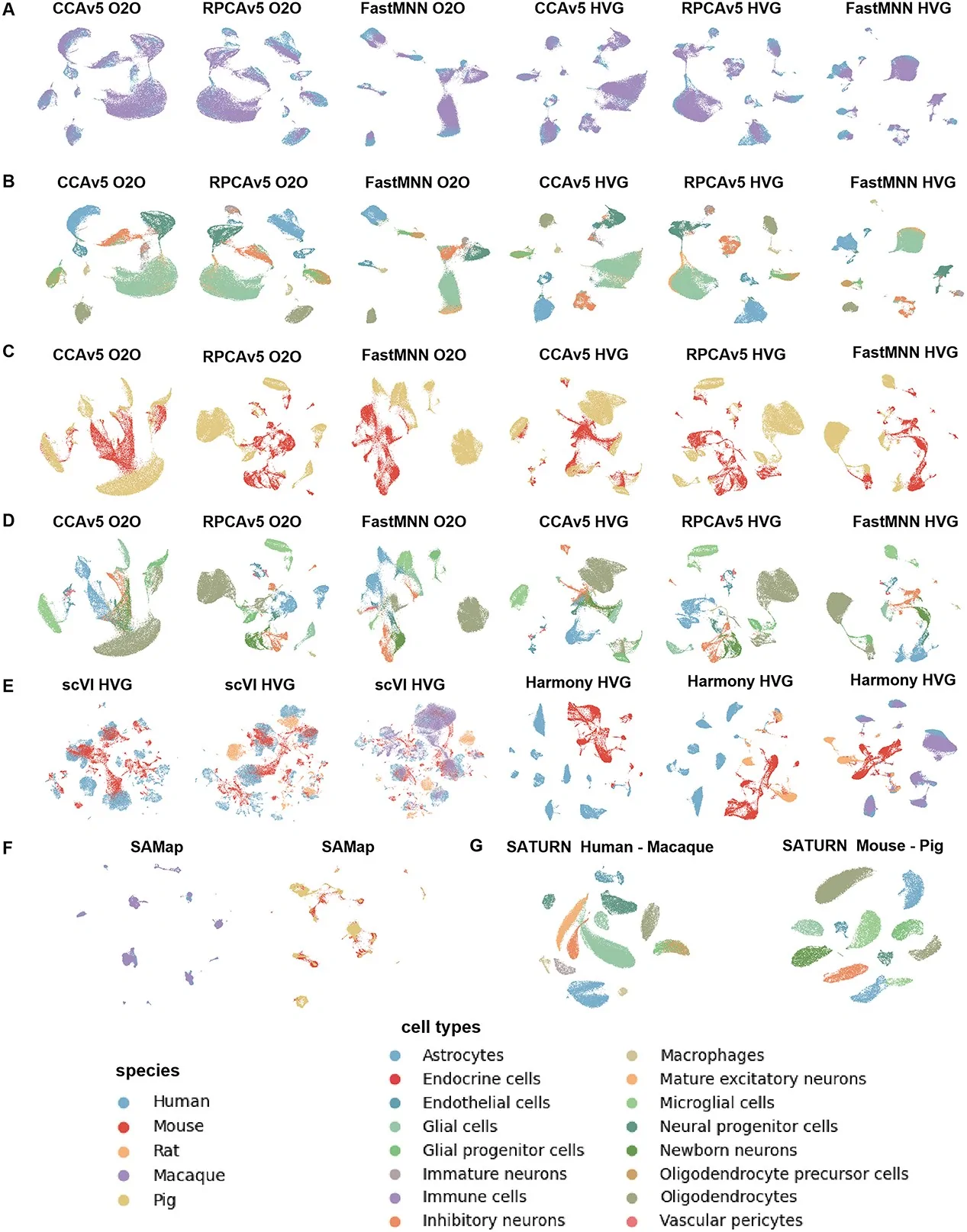
UMAP visualization results of cross-species single-cell transcriptomic data integration; (A and B) UMAP plots of integration tasks for closely related species (human and rhesus macaque), colored by species labels (A) and cell type labels (B); (C and D) UMAP plots of integration tasks for distantly related species (mouse and pig), colored by species labels (C) and cell type labels (D); (E) UMAP plots of multispecies integration tasks, colored by species labels; (F and G) UMAP plots of cross-species integration using SAMap and SATURN.

We visualized the results from all integration tasks to illustrate the cross-species single-cell transcriptomic data integration diversity. The UMAP plots served as the foundation for subsequent quantitative assessment. In closely related species integration, most methods exhibited favorable cell type clustering and species mixing with high overlap, while preserving boundaries (Fig. 2A and B). In contrast, distantly related species integration exhibited pronounced species-specific clustering, fuzzier boundaries, and increased difficulty. Gene selection strategies exerted more pronounced effects on integration quality in distantly related tasks (Fig. 2C and D). In multispecies scenarios, method-specific differences were evident: scVI enhanced species mixing, while Harmony prioritized feature retention (Fig. 2E). SAMap, leveraging sequence similarity, showed a strong cross-species mixing capability (Fig. 2F), whereas SATURN, utilizing protein language models, demonstrated superior cell type structure preservation (Fig. 2G). These results highlight the importance of method selection and underscore the need to establish an objective evaluation framework to quantitatively assess integration quality.

### Construction and application of machine learning evaluation model

**Figure 3:**
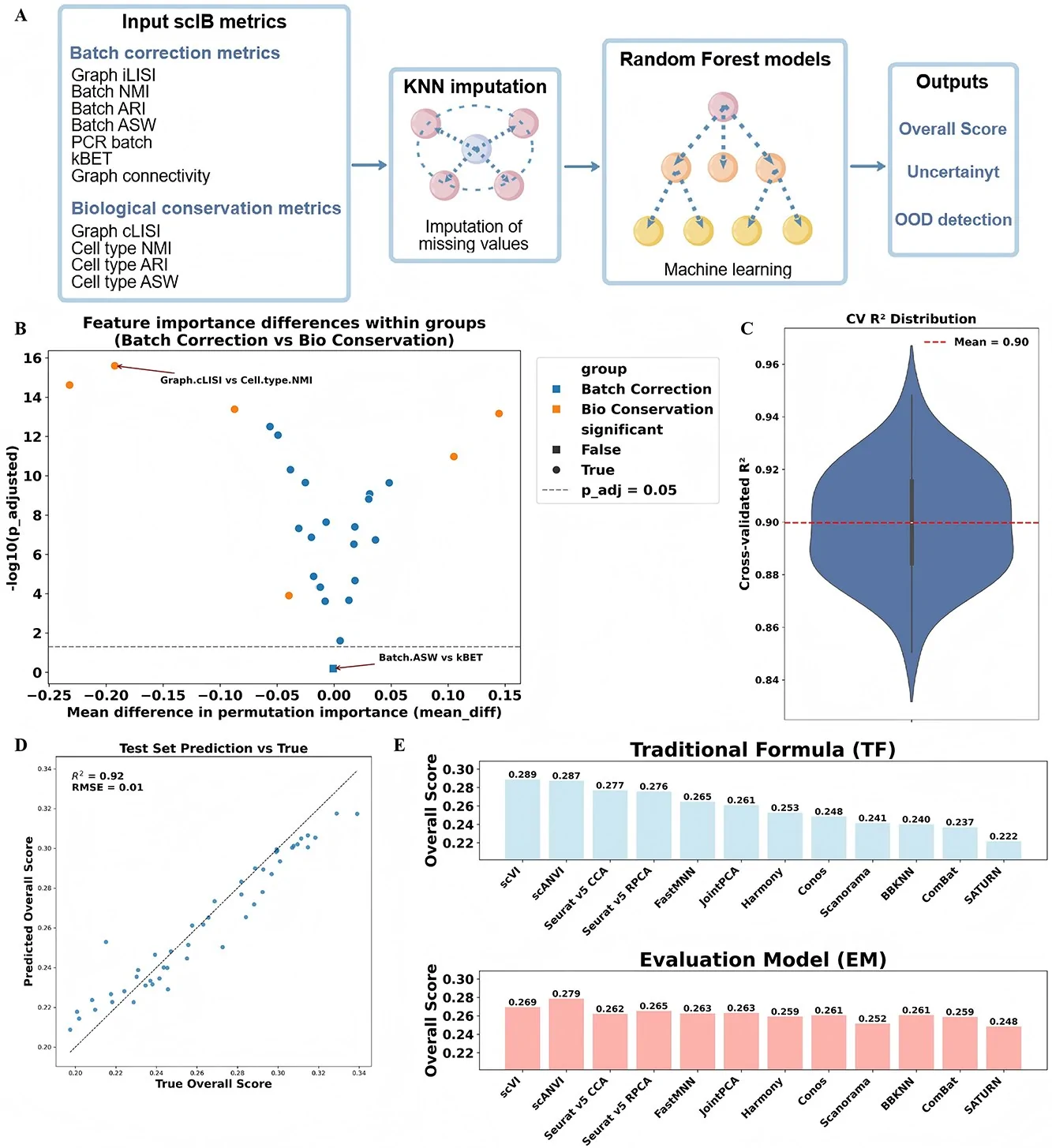
Machine learning evaluation model provides more objective scoring criteria; (A) this diagram was created with BioGDP.com [59] and illustrates the model workflow, including input evaluation metrics, the KNN imputation algorithm, the Random Forest Regressor, and the final outputs of overall score, uncertainty, and outlier detection; (B) we computed permutation importance for the trained RF model on the test set (20 repeats); we then performed pairwise comparisons of features within two groups (batch correction and bio conservation) using paired *t*-tests on the repeated permutation importances; *P*-values were Holm-corrected for multiple testing; a volcano-like plot [mean_diff versus –log10(p_adjusted)] visualizes the pairwise differences, with colors indicating group membership and shapes indicating significance; extreme points (most significant and least significant) are annotated; (C) the violin plot of the cross-validated *R*^2^ distribution shows the *R*^2^ values on the *Y*-axis, which measure the strength of the model’s predictive capability; the bar in the middle represents the interquartile range of the data, while the dashed line indicates the mean *R*^2^ value, suggesting that the overall predictive performance of the model is good; (D) the figure illustrates the relationship between the model’s predicted scores and the true overall scores on the test set; the *X*-axis represents the true overall scores, while the *Y*-axis shows the predicted overall scores; the dots represent the predicted values compared to the actual values for each test sample, reflecting the model’s performance on the test set; the dashed line indicates the ideal 1:1 line; the *R*^2^ value is 0.92, indicating the model’s strong predictive capability on the test set and high fitting quality; the root mean square error is 0.01, indicating that the error between the model’s predictions and the actual data is very small; (E) overall scores for traditional formula (TF) and evaluation model (EM).

In the field of cross-species single-cell transcriptome data integration, the scientific and artificial evaluation of the effectiveness of different integration methods has always been a critical challenge. Traditional evaluation systems often rely on subjective assignments of metric weights based on experience and face issues such as insufficient comparability and difficulty in handling missing values when dealing with different batches, species, or task contexts, making it difficult to fully reflect the true performance of each method. To address this, we developed a data-driven integrated evaluation model, ScCrossScore, based on machine learning (Fig. 3A). This model learns the non-linear mapping relationship between the evaluation metrics and the overall score and employs a data-driven approach to quantify the contribution of each metric, thereby providing a more accurate assessment of integration performance.

Before constructing the evaluation model, we established a systematic evaluation index system with 11 recognized metrics from 2D: batch correction and biological conservation, laying the foundation for model construction. In the initial construction phase, we integrated our team’s evaluation results of 13 integration methods across 27 cross-species tasks, along with publicly available assessment data from other researchers [22], expanding the coverage of methods, tasks, and metrics. To address the missing values for SAMap and another method, we employed the KNN interpolation algorithm for data supplementation. To minimize the introduced bias, we applied MinMaxScaler normalization to all metrics, scaling their values to the range [0, 1], and carefully determined the optimal *k*-value through CV. Additionally, we compared three approaches for handling missing values: data with no missing metrics, imputed data, and data with NA values uniformly set to 0, ensuring that KNN imputation enhanced comparability without introducing extreme biases (Supplementary Figs S1 and S2). We employed Random Forest Regression as the core algorithm to analyze the relationships between multidimensional evaluation metrics and the integrated score, optimizing the parameters to obtain the optimal model configuration for predicting the overall score. To mitigate the potential bias in traditional scoring methods, we conducted permutation importance analysis [59–63] by randomly permuting the values of each individual metric feature and repeatedly calculating the decline in model performance, thereby quantifying the contribution of each evaluation metric (Supplementary Fig. S3). To further elucidate the relative contributions of individual features to the score importance, we conducted permutation importance difference analyses on feature pairs within both the batch correction and bio-conservation groups. For each group, we computed the mean difference in permutation importance for every pair of features across 20 repeats, along with the Holm-corrected *P*-values from the paired *t*-tests. The results revealed multiple pairs with significant differences between the groups. For instance, within the bio-conservation group, cell type NMI and cLISI exhibited the most prominent differences, with cell type NMI demonstrating significantly higher importance than cLISI. Conversely, the difference between Batch.ASW and kBET in the batch-correction group was less pronounced (Fig. 3B). This indicates that assigning equal weights to the metrics within the batch effect correction and biological conservation groups is not equitable; the weighting scheme of this evaluation metric model is more refined than that of traditional formulas. We also validated the other capabilities of the model. The model performed excellently on the 85% training set (*R*^2^ > 0.95) and maintained a robust predictive capability on the 15% independent test set (*R*^2^ = 0.89). Through repeated K-fold CV, we further confirmed the model’s generalization ability, with the average *R*^2^ value remaining stable within the confidence interval, indicating that the model predictions were statistically reliable (Fig. 3C). Regarding error characterization, the mean absolute error and root mean square error of the test set were within reasonable ranges. The residual scatter plot did not show a systematic structure, with the residuals exhibiting a nearly symmetrical distribution, indicating no significant deviations in the model fitting (Fig. 3D).

Finally, we applied the model to the independent test dataset, computed new scores, and compared them with the original scores to assess the method performance under the two scoring systems. Several methods showed different rankings (Fig. 3E). scVI and scANVI remained top-ranked in both the traditional and new evaluation systems, whereas Seurat v5 CCA, Harmony, and Scanorama dropped significantly. Seurat v5 RPCA, JointPCA, and BBKNN showed slight ranking improvements. This may be because scVI and scANVI, as deep learning models, capture complex biological signals and batch variation better, adapting more flexibly to the new evaluation framework. In contrast, Seurat v5 CCA, Harmony, and Scanorama focused more on batch effect removal, whereas Seurat v5 RPCA, JointPCA, and BBKNN prioritized biological feature preservation (Supplementary Table S5), leading to ranking shifts under the refined weighting scheme. Moreover, SAMap and SATURN, which previously scored low due to NA values in traditional evaluations, also achieved improvement.

### Systematic assessment of factors affecting cross-species data integration

**Figure 4:**
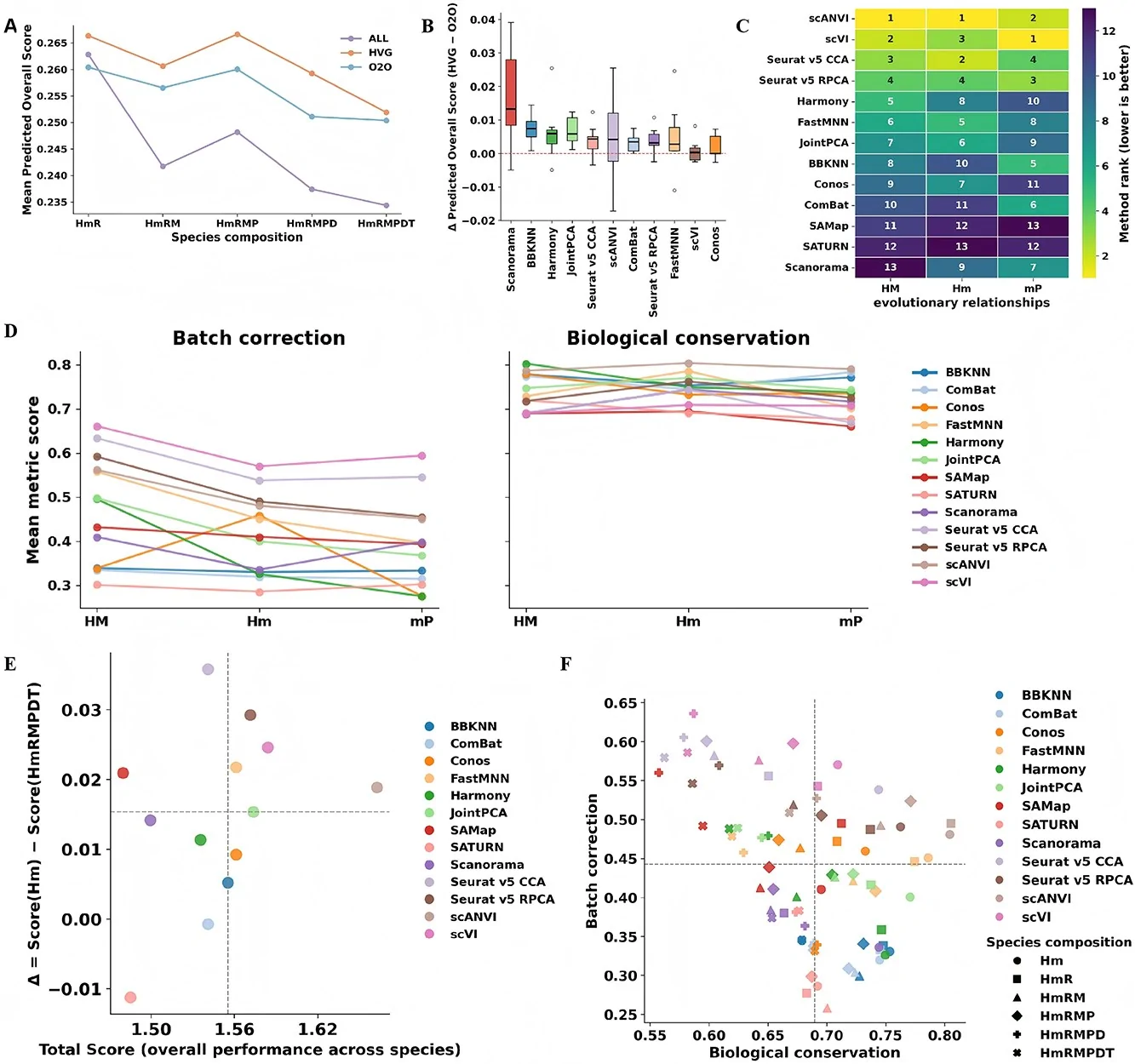
Impacts of gene selection, evolutionary relationships, and species number on cross-species integration; (A) integration performance using different gene strategies across various cross-species combinations; (B) the stability and effectiveness of methods for different gene strategies; distribution of overall score differences (HVGs−O2O) for each method across cross-species integration tasks; red dashed line marks zero (no difference); positive values indicate HVGs outperform O2O; boxes show interquartile range, center lines show medians, and whiskers represent data range with outliers displayed as individual points; (C) a heatmap showing the ranking changes of integration methods under different evolutionary relationships; the *y*-axis represents various integration methods, while the *x*-axis indicates the evolutionary relationships between species: closely related (HM), intermediate evolutionary distance (hm), and distantly related (mP); the shade of color represents the rank, with lighter colors indicating better rankings for the methods; (D) changes in average scores of various integration methods in the batch correction and biological conservation metrics under different evolutionary relationships; the *x*-axis represents the evolutionary relationships between species: closely related (HM), intermediate evolutionary distance (hm), and distantly related (mP); the *y*-axis indicates the corresponding average scores; each line corresponds to an integration method and is differentiated by color; (E) the figure illustrates the relationship between total score (overall performance across species) and Δ (the score difference between hm and HmRMPDT) for different cross-species integration methods; the *x-*axis represents the total score values, while the *y*-axis indicates the Δ values; each point represents a specific integration method, and the figure highlights the median values of total score and Δ, with dashed lines for reference; (F) the figure illustrates the relationship between biological conservation (*x*-axis) and batch correction (*y*-axis) for different cross-species integration methods; each point represents a specific integration method, with colors indicating different methods and shapes representing various species combinations; the figure also displays dashed lines for the median values of biological conservation and batch correction for reference and comparison.

The main challenge in cross-species integration is the distinct gene sets across species [64], and different gene selection approaches alter data characteristics, dimensionality reduction effects, integration outcomes, and biological analysis reliability. To assess how gene selection impacts integration, we compared three strategies: O2O focuses on cross-species orthologs to reduce genomic interference; HVGs retain informative, variable genes to capture shared features; and ALL offers a comprehensive perspective via full gene integration. Using 13 integration methods, we performed 216 integrations, evaluated them with 11 metrics, and scored them using our constructed model (Supplementary Table S6). By calculating the mean score of all methods across different gene selection strategies, we found that the HVGs consistently achieved the highest scores across all species combinations (Fig. 4A). We analyzed the score differences between the HVGs and O2O strategies for each method (Fig. 4B). Scanorama showed improved HVG scores, but high volatility. The scANVI achieved the highest scores under both strategies, with higher HVG scores but some instability. The scVI ranked second in both scenarios, with stable scores, despite no significant improvement. BBKNN, which performed poorly with O2O, showed notably improved scores, rankings, and stability with the HVGs.

Evolutionary distance is a core factor influencing the similarity and divergence of gene expression across species and may therefore directly determine integration difficulty and performance ceiling. In this study, human and rhesus macaque, both of which belong to the order Primates, with high overall genomic similarity, can serve as the closest evolutionary relationship for integration tasks. Mouse and human, belonging to different orders but sharing Euarchontoglires in mammalian evolution, can be used as intermediate evolutionary relationship integration tasks. Although mouse and pig also belong to different orders, pig is classified as Laurasiatheria, which is more distantly related to human than to mouse. Therefore, mouse and pig were selected as the most distantly related integration tasks using 13 methods to integrate tasks across three evolutionary levels, analyzed results with 11 metrics, and generated scores via our evaluation model (Supplementary Table S7). Overall, closer evolutionary relationships allowed for broader selection of methods (Supplementary Fig. S4). The top four methods (scANVI, scVI, Seurat v5 CCA, and Seurat v5 RPCA) maintained stable performances across all levels. Scanorama and ComBat showed rising rankings with increasing evolutionary distance. SAMap and SATURN had stable integration capabilities that were unaffected by evolutionary changes (Fig. 4C). To explain score variations, we grouped methods based on evolutionary relationships and calculated their mean scores for batch correction and biological conservation (Fig. 4D). scANVI benefited from the higher weighting of biological conservation; scVI remained top performing with strong batch correction. Scanorama’s batch correction and biological conservation of ComBat improved with distance, while the overall scores of Harmony, FastMNN, and JointPCA decreased because of weakened batch correction.

We divided our cross-species integration tasks into six based on the number of species, and applied 13 methods to each. The outcomes were evaluated using 11 metrics and scored using our model (Supplementary Table S8). All methods achieved the highest scores with five species; scores declined when species exceeded five, and disparities among methods narrowed to seven species. scANVI ranked first consistently, but declined noticeably after five species, dropping to an average of seven species. The Seurat v5 CCA and Conos scores decreased from the start, whereas SATURN improved gradually (Fig. 4E). Among the seven species (HmRMPDT), including human, mouse, rat, rhesus macaque, pig, dog, and tree shrew (HmRMPDT), only scANVI maintained a strong performance in both evaluation dimensions, with UMAP plots showing good species merging and cell type clustering (Supplementary Fig. S5). JointPCA consistently achieved stable scores owing to its sustained high biological conservation capability during the early stages; however, after exceeding five species (HmRMP), its biological conservation capability declined sharply, shifting toward batch correction. Although it showed some overcorrection, it still performed relatively stable integration capabilities overall compared to other methods. In contrast, the Seurat v5 CCA showed a continuous decline in biological conservation capability after exceeding two species (Hm), completely favoring batch correction. Consequently, while it consistently showed good merging in the species label UMAP plots, the actual correction process led to reduced biological interpretability and cell type mixing (Fig. 4F and Supplementary Fig. S6).

### Development of a cross-species integration assessment website platform

**Figure 5:**
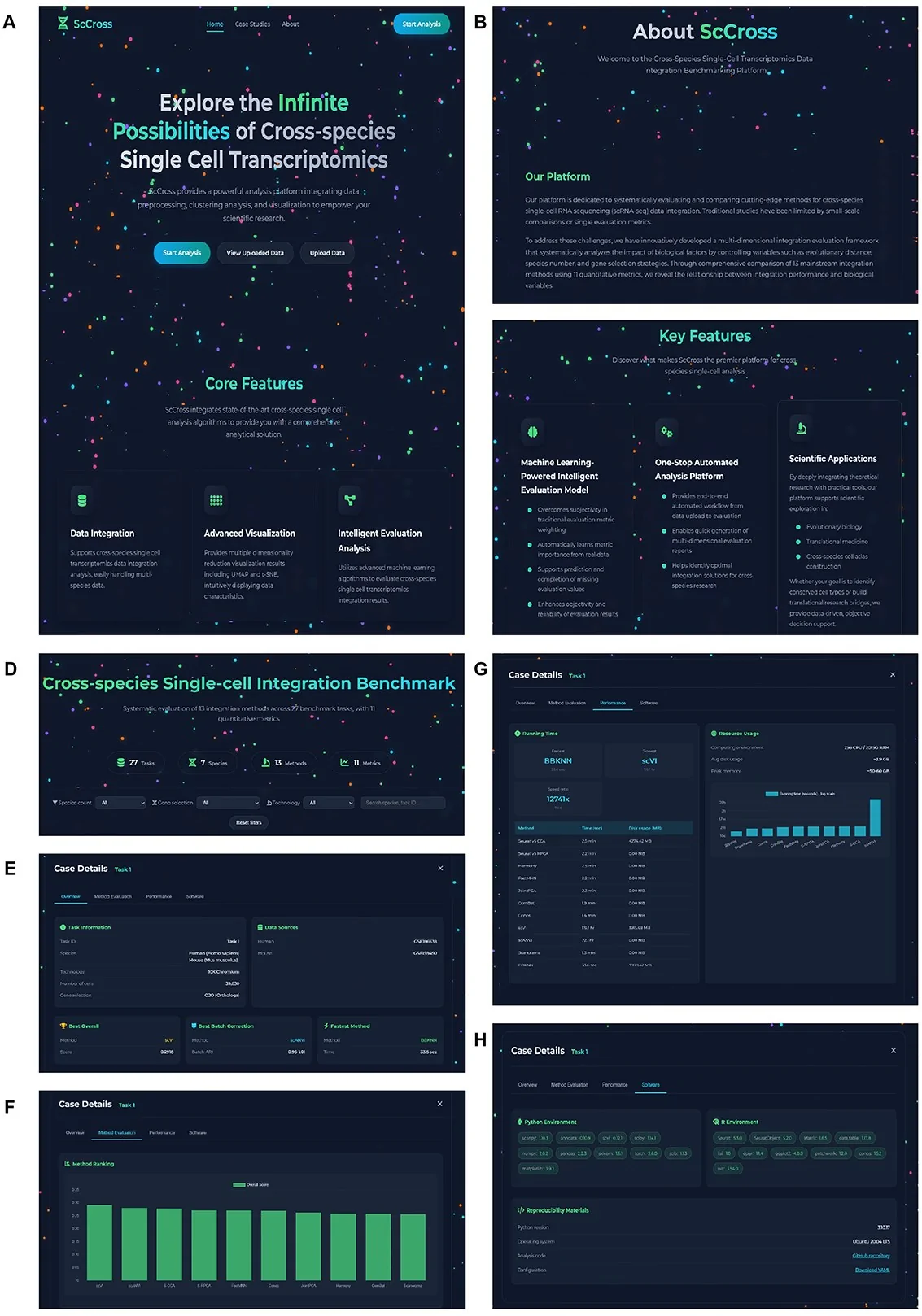
Main interfaces and usage of ScCross; (A) ScCross platform homepage showing the tagline and core operation options: “Start analysis,” “View Uploaded Data,” and “upload data”; (B) about page of the ScCross platform, detailing its evaluation framework, models, pipelines, and applications in cross-species research, along with version information and service commitment; (C) privacy policy, data availability, and code availability statements; (D) benchmark summary; (E) case study library; comprehensive metadata for task 1 (human–mouse integration): 10× chromium technology, 39 630 cells, ortholog gene selection strategy (O2O), and data source accession numbers (GSE186538 for human, GSE158450 for mouse); (F) method rankings; overall score ranking of integration methods based on 11 quantitative metrics; (G) running time and resource usage; (H) software environment; detailed software environment for reproducibility, including python packages and R packages.

ScCross is a cross-species single-cell evaluation platform tailored for biomedical research. It streamlines intricate bioinformatics workflows, enabling users without programming expertise to conduct professional single-cell analysis. It comprises three core modules: data integration, advanced visualization, and intelligent assessment. The homepage features tag lines and core functional entries. Once users upload qualified, properly formatted data (file size, file format, and whether it includes cell annotations) and select the preferred integration methods, ScCross automatically completes the analysis and feeds the evaluation results back. The Introduction page elaborates on its evaluation framework, machine learning (ML)-based assessment model, automated pipeline, and cross-species research value. A benchmark case library is also provided to detail task-specific information and method performance. On the task filtering page, users can rapidly screen for optimal cross-species single-cell transcriptomic integration methods using multiple custom conditions (Fig. 5).

To validate the usability of the platform, we integrated and evaluated a dataset of peripheral blood mononuclear cell (PBMC) samples from human, mouse, and dog [65]. The results demonstrated that scANVI and scVI outperformed the dataset. CCA, the method employed in studies utilizing these PBMC samples, was also ranked among the top performers in the evaluation (Supplementary Table S9 and Supplementary Fig. S7). These results indicate that the evaluation framework can maintain strong performance in non-conservative and highly heterogeneous biological contexts.

## Discussion

In this study, 13 mainstream integration methods were systematically evaluated under various conditions of gene selection, evolutionary relationships, and species number. We developed ScCrossScore, a machine-learning-based evaluation model, to enable data-driven assessment. The results showed that prioritizing HVGs based on one-to-one orthologs improved the integration performance. scANVI, scVI, Seurat v5 CCA, and Seurat v5 RPCA performed stably across evolutionary levels, with scANVI showing clear advantages in multispecies integration. Finally, we developed ScCross, a platform for cross-species single-cell integration analysis, to support the method selection and interpretation of results.

**Figure 6:**
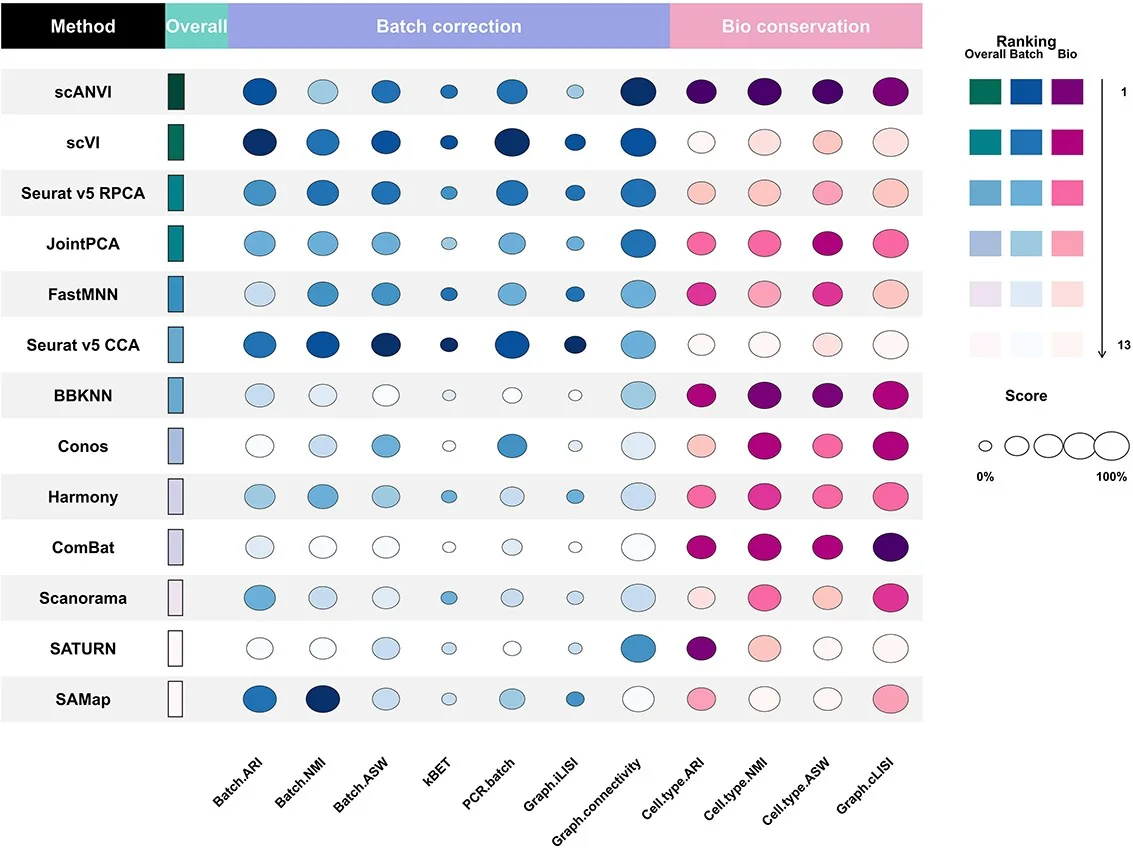
The average overall scores and ranking of all methods in different cross-species integration tasks; this is a rating heat-map where methods with higher overall scores are ranked higher, and a darker color indicates a higher overall score; for detailed metrics, a higher score also corresponds to a darker color; the size of the circle represents the relative magnitude of the score for each specific metric, with a larger circle indicating a relatively higher score for that metric within the corresponding method.

This study presents a machine-learning-based evaluation model. Compared to traditional approaches, it provides a more data-driven framework for metric weighting and missing data processing. We found that biological conservation metrics were generally more important than batch-effect removal metrics, which is consistent with prior research. Model application revealed ranking shifts of integration methods under the new scoring framework (Fig. 6 and Supplementary Fig. S8). In particular, scVI and scANVI, which are built on deep learning frameworks, capture complex biological signals and batch differences to achieve high performance. As a semi-supervised model, scANVI utilizes cell labels during training. To avoid potential unfairness in the comparison of biological conservation resulting from this, we conducted a detailed comparison across metrics that are label-dependent and label-independent (Supplementary Fig. S9), demonstrating that the high scANVI score is attributable to the combined contributions from both categories of metrics. Meanwhile, the classical methods, Seurat v5 RPCA and JointPCA, show stable and favorable performance. The Seurat v5 CCA works well on closely related and small datasets but ranks poorly overall, as it overcorrects batches at the expense of biological retention. SAMap and SATURN also achieved improved scores, proving our model’s strength in missing data handling and optimized weighting.

In cross-species integration tasks, the quality of gene selection is often more critical than the number of genes chosen. Regardless of evolutionary relationships or species diversity, prioritizing the selection of HVGs based on one-to-one orthologs for integration not only ensures the comparability of features across species through orthologous relationships, but also retains the most distinguishing biological characteristics of cell types through variability selection. This approach not only enhances the speed of integration but also reduces noise caused by genomic differences, thereby improving data comparability and integration performance.

Through scoring analysis, we found that species with closer evolutionary relationships allowed a wider choice of integration methods and achieved higher scores, consistent with the UMAP visualizations (Supplementary Fig. S10). Biological similarity improves integration performance. Methods including scANVI, scVI, Seurat v5 CCA, and Seurat v5 RPCA were performed stably across evolutionary distances. SAMap and SATURN, which use gene sequences or protein embeddings, are less affected by evolutionary divergence, showing strong stability and flexibility for cross-species integration.

In cross-species single-cell integration, the number of species strongly affects the integration complexity, analysis accuracy, and model stability. All the methods achieved the highest scores when integrating the five species. As the species number increased, the scores generally declined, and inter-method differences narrowed. scANVI performed well in multispecies integration, whereas JointPCA and BBKNN also showed advantages owing to their stable performance. SATURN appeared to be more suitable for larger species sets. In contrast, Seurat v5 CCA performed notably worse with more species, likely because of overemphasis on batch correction at the cost of biological features.

To facilitate applications and improve usability, we developed a cross-species single-cell integration evaluation platform, ScCross. It supports fully automated data uploads, quality control, integration, machine learning scoring, and report generation. With a modular design and friendly interface, it accepts common formats and mainstream methods, enabling a one-stop evaluation and method recommendation. This lowers the research barrier and provides an efficient tool for cross-species single-cell analysis, thereby promoting the development of integration methods.

In practical cross-species studies, integration efficiency and adaptability should also be considered, in addition to gene selection, evolutionary relationships, and species number. As the methods vary greatly in terms of computing cost and scalability, we performed relevant tests. For imbalanced data, SAMap showed strong robustness in avoiding the dominance of small samples (Supplementary Fig. S11). Traditional methods such as Seurat v5 CCA, RPCA, and JointPCA feature simple preprocessing and high efficiency on small-to-medium data, making them suitable for rapid analysis (Supplementary Table S10). While the deep learning methods scVI and scANVI performed excellently, SAMap and SATURN also showed potential. However, all require considerable computing resources, and the efficiency drops sharply with large amounts of data. SAMap is time-consuming in BLAST and homology preprocessing (Supplementary Fig. S12); meanwhile, SATURN requires extra protein and gene processing. Thus, large-scale applications must balance integration performance and computational cost.

Overall, this study systematically compared mainstream cross-species single-cell integration methods and provided novel methodological insights. Through multitask design, a machine learning evaluation framework, and a visual platform, it establishes a standardized, intelligent, and reproducible workflow for cross-species data integration. The ScCrossScore model offers a new perspective on missing data imputation, weight learning, and quantitative scoring. Combined with the ScCross web platform, this supports efficient method selection and application. However, this study focuses on the hippocampus of mammals because of its significance and broad application in various scientific research fields, allowing for effective comparison and analysis of rich data resources. Nevertheless, although this choice provides profound biological insights, it limits the generalizability of the results, as single-cell data from different tissues, species, developmental stages, or physiological conditions may exhibit distinct characteristics and challenges during integration. Therefore, future research should consider expanding the scope of integration analysis to include single-cell transcriptomic data from other regions, tissues, or different developmental stages to validate the applicability and precision of the proposed methods across diverse biological contexts. Furthermore, considering that in practical integrative studies, more complex integration scenarios may cause core parameters and cell annotations to affect the integration performance of different methods, we will further enhance parameter selection and the integration of cell annotations to improve the robustness of our model and platform when dealing with complex cross-species heterogeneity. Moreover, we will develop a decision tree framework to support long-term progress in single-cell and evolutionary biology research.

**Key Points**

- We systematically compared 13 mainstream cross-species single-cell integration methods using 11 metrics across 27 tasks and quantified how gene selection, evolutionary relationships, and species number affect integration performance.
- We constructed ScCrossScore, a machine learning-based evaluation model that improves objectivity, enhances method comparability, and enables the robust handling of missing values in integration assessment.
- We developed the user-friendly online platform ScCross for automated evaluation and scenario-specific method recommendation, which greatly lowers the barrier for researchers without a bioinformatic background.

## Supplementary Material

Supplementary_Figures_bbag490

Supplementary_Tables_bbag490

HiC_BIB_R1_suppl_bbag490

## Data Availability

Datasets were collected from published studies as described in Supplementary Tables S1 and S2. The preprocessed datasets for each task are available at https://doi.org/10.6084/m9.figshare.31968882. The source code and corresponding step-by-step manual are available at https://doi.org/10.6084/m9.figshare.31976259. ScCross is freely available online at https://sccross.pythonanywhere.com/.
