# Supplementary material for: Systematic benchmarking and optimal strategy selection of cross-species integration methods
Source: Brief Bioinform. 2026 Sep 10;27(5):bbag490. doi: 10.1093/bib/bbag490 (PMC13561306; doi:10.1093/bib/bbag490)
Supplement: Supplementary file 1 [file hic_bib_r1_suppl_bbag490.pdf]

Hi-C Informed Kernel Association Test (HiC-KAT) for Integrating 3D Genome Structure into Variant-Set Analysis

Yueyang Huang[1], Riddhik Basu[2], Yuhuan Cheng[1], Wenbin Lu[2], Shannon T. Holloway[3], Chang Chen[4], Yun Li[4,5], and Jung-Ying Tzeng[*1,2]

[1]Bioinformatics Research Center, North Carolina State University, Raleigh, NC, USA
[2]Department of Statistics, North Carolina State University, Raleigh, NC, USA
[3]Department of Population Health Sciences, Duke University, Durham, NC, USA
[4]Department of Biostatistics, University of North Carolina at Chapel Hill, Chapel Hill, NC, USA
[5]Department of Genetics, University of North Carolina at Chapel Hill, Chapel Hill, NC, USA

[*]Corresponding author: Jung-Ying Tzeng, Department of Statistics and Bioinformatics Research Center, North Carolina State University, Raleigh, NC, USA. Email: jytzeng@ncsu.edu, Phone: +1-919-513-2723.

Supplemental Figures

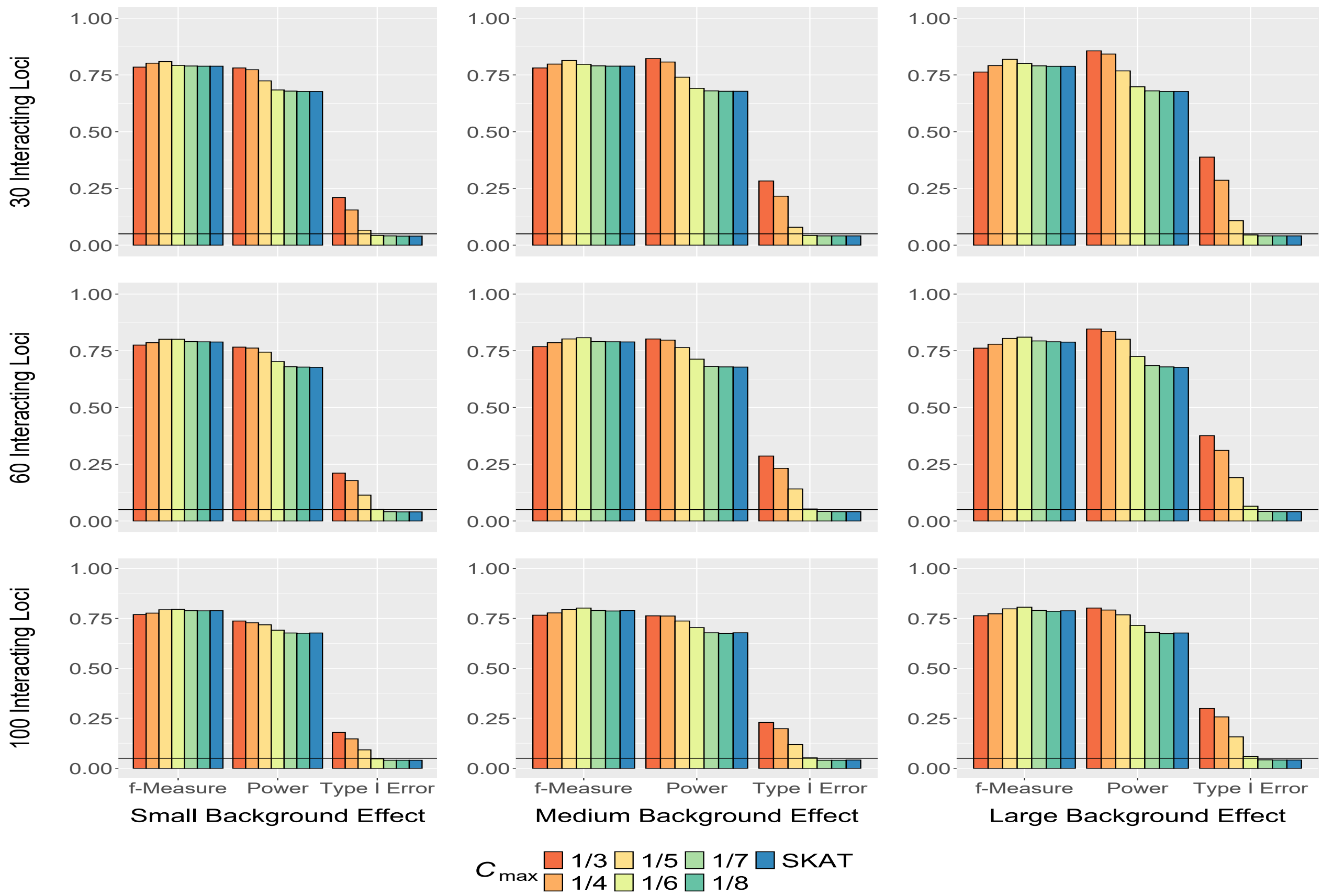

Figure S1. Simulation results for sample size $n = 9,000$ showing type I error rate, power, and f-measure. Type I error rate is calculated when the target locus has no causal effect but its interacting loci do (i.e., Setting II of the type I error Simulation). Power is calculated when both the target locus and its interacting loci have causal effects.

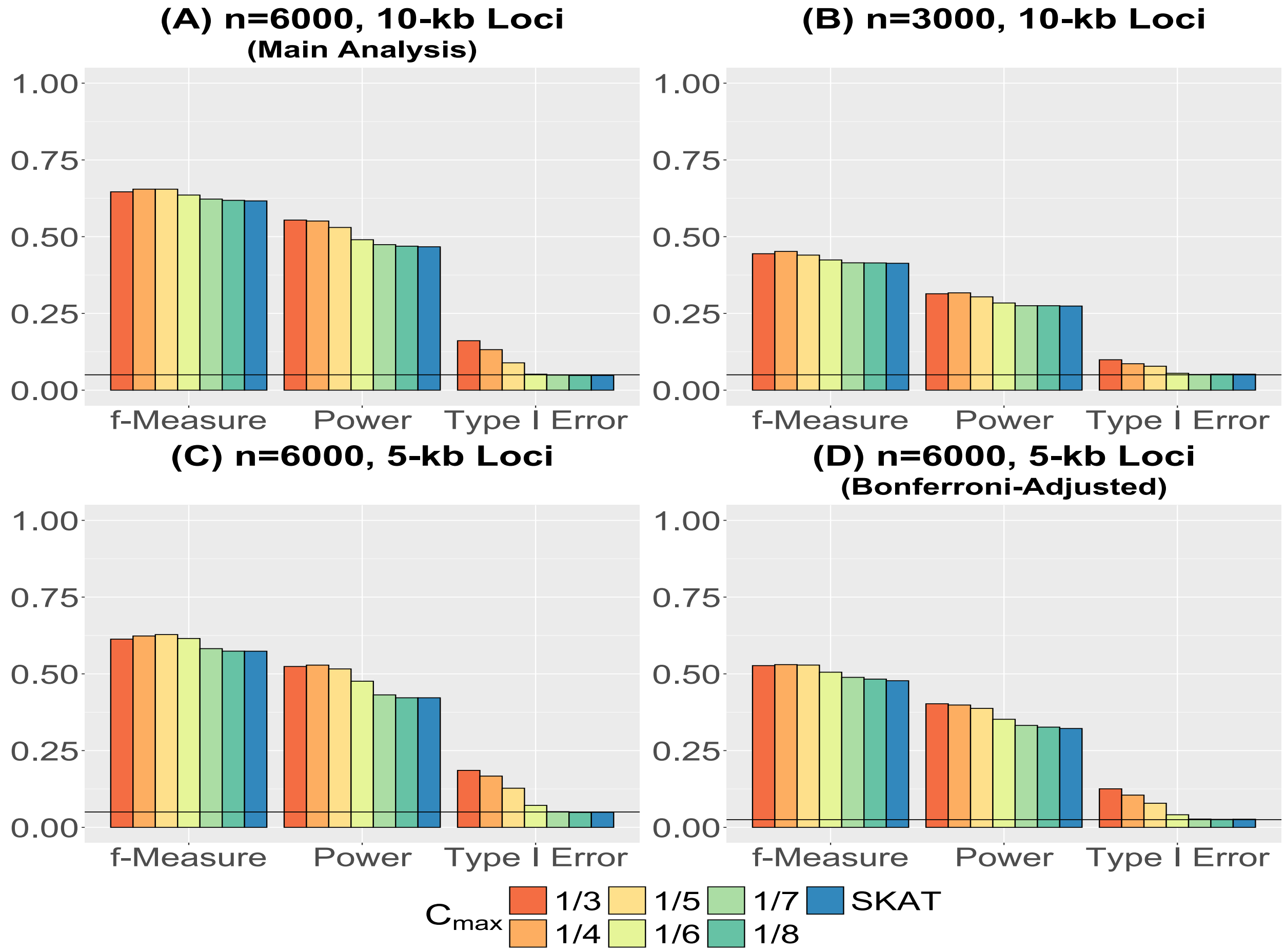

Figure S2. Performance of HiC-KAT across different sample sizes and locus resolutions. Simulations are conducted based on the chr5 setting under a medium background effect with 60 interacting loci. (A) Main analysis with n=6,000 and 10-kb loci. (B) Reduced sample size (n=3,000) with 10-kb loci. (C) Finer locus resolution (5-kb loci) with n=6,000. (D) Results for 5-kb loci with Bonferroni adjustment. Performance is evaluated in terms of f-measure, power, and Type I error rates across different c_{max} values using 1000 simulation replicates.

Supplemental Tables

	Minimum	1st Quartile	Median	3rd Quartile	Maximum
**(A) Number of interacting loci per locus**					
Whole Genome	1	27	64	100	185
Chromosome 5	1	30	68	104	180
**(B) $\sqrt{Z_{\ell\ell'}}$ for locus pairs within the same chromosome**					
Whole Genome	1.401	1.709	2.066	2.588	6.185
Chromosome 5	1.401	1.710	2.068	2.589	6.185
**(C) $\sqrt{Z_{\ell\ell'}}$ for locus pairs within ± 1 Mb**					
Region selected	1.401	1.783	2.279	2.851	6.185
Chromosome 5	1.401	1.818	2.260	2.862	6.185

Table S1. Comparisons of Hi-C features between whole-genome and chromosome 5 data based on (A) the number of interacting loci per locus, (B) $\sqrt{Z_{\ell\ell'}}$ within a chromosome, and (C) $\sqrt{Z_{\ell\ell'}}$ within ± 1 Mb. Loci ℓ and $\ell^{'}$ are considered as interacting if their Hi-C contact q-value $q_{\ell\ell'} < 0.05$. $\sqrt{Z_{\ell\ell'}} = \sqrt{\Phi^{-1}\left(1 - \frac{q_{\ell\ell'}}{2}\right)}$.

	$c^*_{\max}$				
α	1/5	1/6	1/7	1/8	SKAT
0.05	0.04482	0.04396	0.04441	0.04443	0.04438
0.01	0.00949	0.00925	0.00921	0.00923	0.00920
0.005	0.00482	0.00475	0.00467	0.00469	0.00469
0.001	0.00094	0.00091	0.00091	0.00092	0.00092
0.0005	0.00044	0.00043	0.00044	0.00044	0.00044
0.0001	0.00006	0.00006	0.00006	0.00006	0.00007

Table 1: Table S2. Type I error rates in Simulation Setting I, evaluated under a medium background effect with 60 interacting loci and $n = 6,000$, based on 10^5 simulation replicates. * : c_{max} indicates the largest values considered in the grid $\mathcal{C} = \{1/100, 1/8, 1/7, 1/6, 1/5, \cdots, c_{\max}\}$.

Table S3. Performance comparisons for full grid $\mathcal{C} = \{1/5, 1/6, 1/7, 1/8, 1/100\}$ vs. reduced grid $\mathcal{C} = \{1/5, 1/6, 1/7, 1/8, 1/100\}$

(A) Chr5 simulation with $n = 6000$

# of Interacting Loci		Small Background Effect		Medium Background Effect		Large Background Effect	
		Full Grid grid	Reduced Grid grid	Full Grid grid	Reduced Grid grid	Full Grid grid	Reduced Grid grid
30	f-measure	0.640	0.644	0.648	0.653	0.656	0.660
	Power	0.497	0.503	0.509	0.515	0.520	0.528
	Type I Error	0.057	0.059	0.061	0.063	0.066	0.073
60	f-measure	0.648	0.647	0.655	0.658	0.666	0.667
	Power	0.514	0.517	0.530	0.537	0.550	0.558
	Type I Error	0.072	0.082	0.089	0.094	0.102	0.116
100	f-measure	0.630	0.629	0.632	0.633	0.637	0.641
	Power	0.490	0.491	0.498	0.502	0.510	0.518
	Type I Error	0.066	0.069	0.077	0.084	0.091	0.099

(B) Chr5 simulation with $n = 9000$

# of Interacting Loci		Small Background Effect		Medium Background Effect		Large Background Effect	
		Full Grid grid	Reduced Grid grid	Full Grid grid	Reduced Grid grid	Full Grid grid	Reduced Grid grid
30	f-measure	0.809	0.809	0.814	0.817	0.819	0.820
	Power	0.724	0.728	0.740	0.750	0.768	0.778
	Type I Error	0.066	0.072	0.079	0.087	0.108	0.120
60	f-measure	0.801	0.801	0.802	0.803	0.804	0.800
	Power	0.744	0.747	0.764	0.775	0.801	0.806
	Type I Error	0.114	0.119	0.141	0.156	0.191	0.209
100	f-measure	0.793	0.789	0.794	0.793	0.798	0.795
	Power	0.718	0.720	0.737	0.741	0.768	0.776
	Type I Error	0.092	0.105	0.119	0.129	0.157	0.175

(C) Chr5 simulation with $n = 3000$
(medium background effect & 60 interacting loci)

	Full Grid	Reduced Grid
f-measure	0.440	0.443
Power	0.304	0.308
Type I Error	0.078	0.081

(D) Chr19 simulation with n=6000

	LCL		LV	
	Full Grid	Reduced Grid	LV Full Grid	Reduced Grid
f-measure	0.618	0.622	0.583	0.573
Power	0.471	0.476	0.430	0.420
Type I Error	0.053	0.054	0.046	0.046
