# Supplementary material for: Systematic benchmarking and optimal strategy selection of cross-species integration methods
Source: Brief Bioinform. 2026 Sep 10;27(5):bbag490. doi: 10.1093/bib/bbag490 (PMC13561306; doi:10.1093/bib/bbag490)
Supplement: Supplementary file 2 [file supplementary_figures_bbag490.pdf]

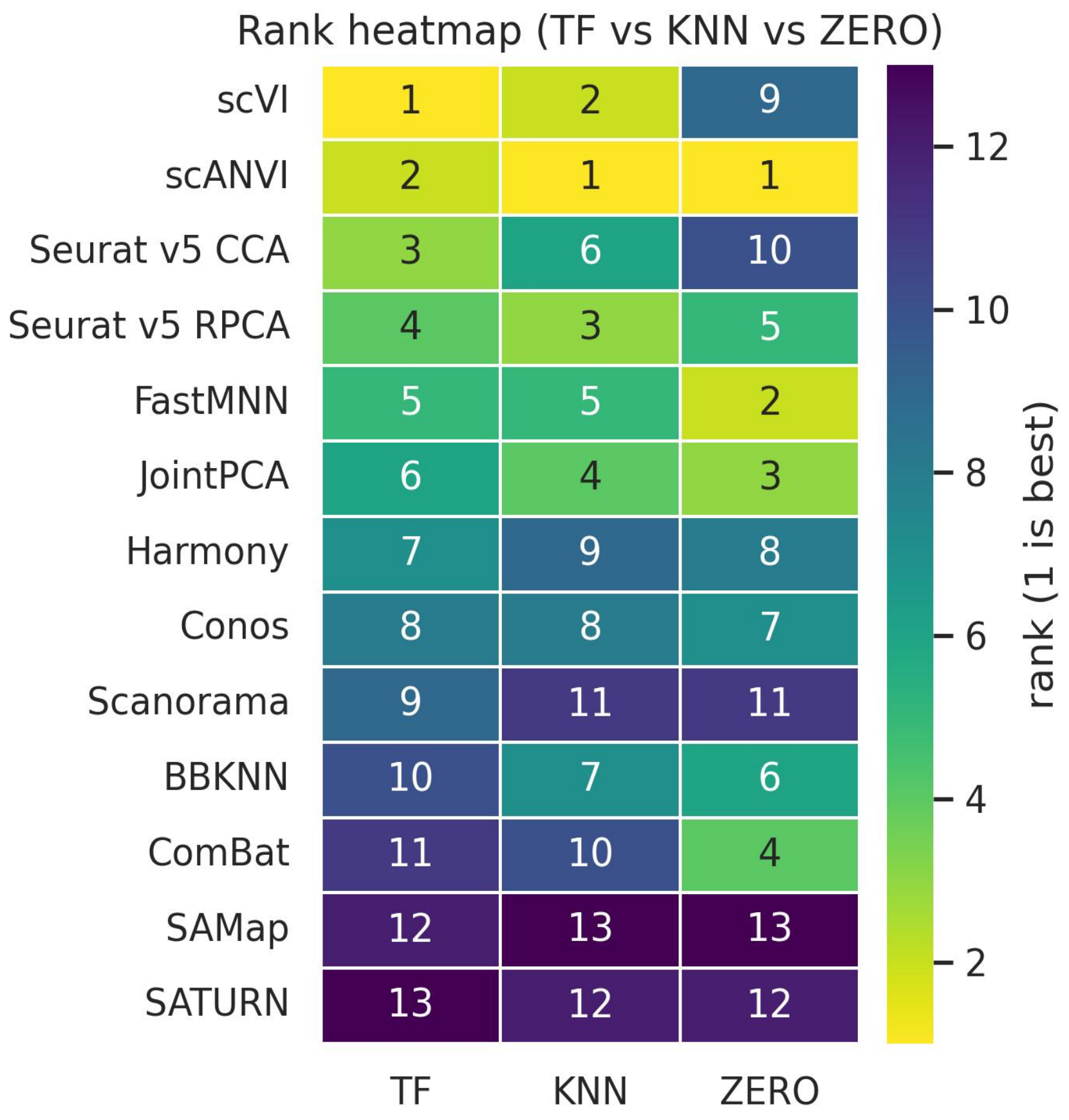

This figure is located in the construction and application of the machine learning evaluation model subsection of the results section.

Figure S1: Evaluation of the KNN-based imputation method and different missing-value handling strategies.We used three missing-value handling strategies, including complete data without missing metrics (TF), KNN-imputed data (KNN), and data with all NA values uniformly set to 0 (ZERO). By comparing the method rankings obtained under these three strategies, we found that simply setting missing values to 0 leads to clearly biased or extreme results.

Alt text: This figure presents a comparative heatmap of method rankings under three missing-value handling strategies: complete data without missing metrics (TF), KNN-imputed data (KNN), and data with NA values uniformly set to 0 (ZERO). The results show that the metric distribution after KNN imputation is most similar to that of the complete data, whereas uniformly setting missing values to 0 leads to biased or extreme metric values.

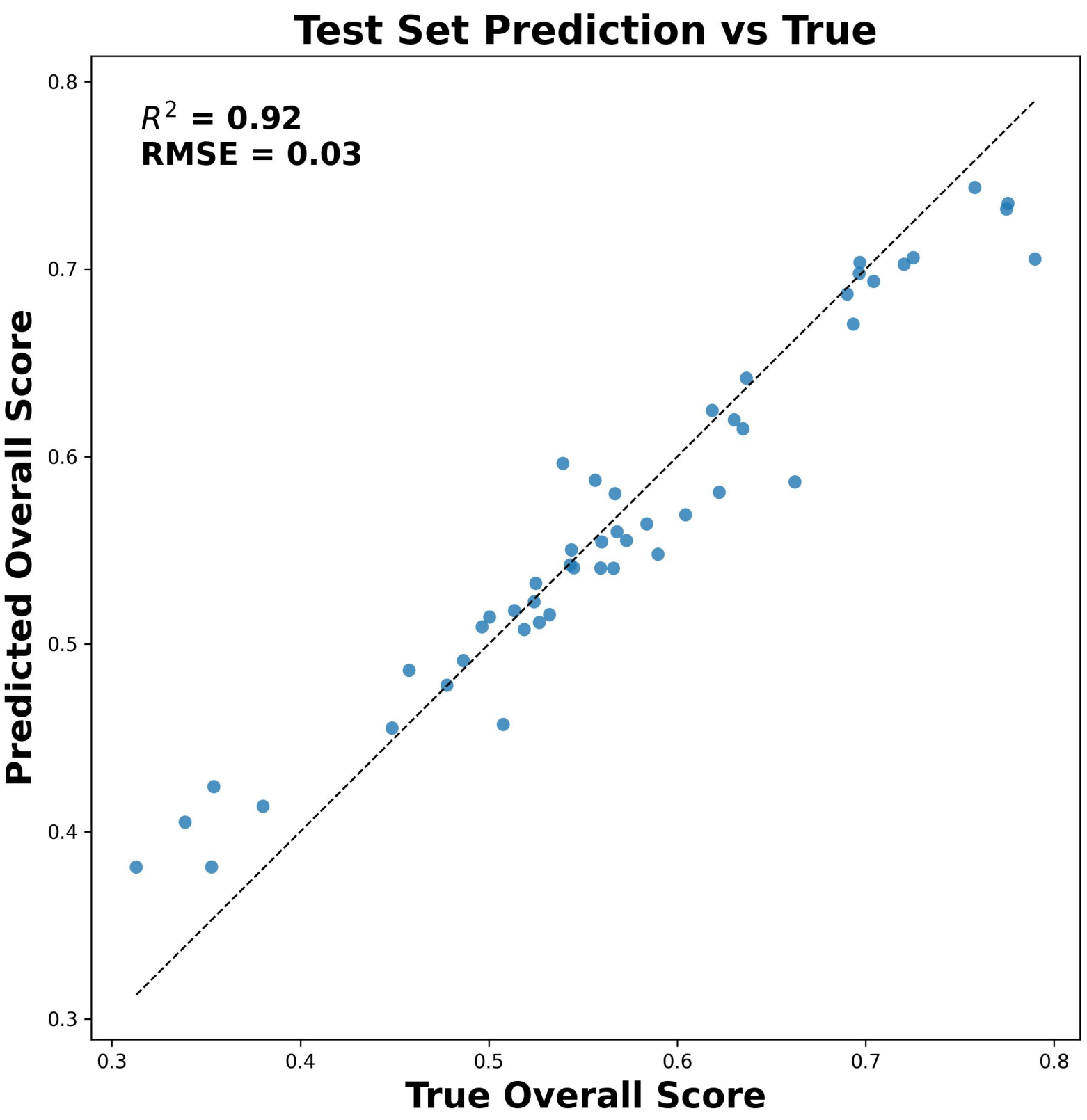

This figure is located in the construction and application of the machine learning evaluation model subsection of the results section.

Figure S2: This figure shows the relationship between the model-predicted scores and the true overall scores on the test set when NA values were uniformly set to 0. The X-axis represents the true overall scores, while the Y-axis shows the predicted overall scores. The blue dots represent the predicted values compared to the actual values for each test sample, reflecting the model's performance on the test set. The dashed line indicates the ideal 1:1 line. The R^2 value is 0.92, the root mean square error (RMSE) is 0.03, indicating that, under this setting, the discrepancy between the model predictions and the actual data is larger than when using KNN imputation.

Alt text： A scatter plot titled "Test Set Prediction vs True" comparing predicted and actual scores. The x-axis represents the "True Overall Score" and the y-axis represents the "Predicted Overall Score." Blue dots show a positive correlation, clustering around a dashed 1:1 line. Text on the chart indicates the R^2 value of 0.92 and RMSE of 0.03. The plot illustrates the model's performance when missing values are set to 0, indicating a larger bias compared to the KNN imputation method.

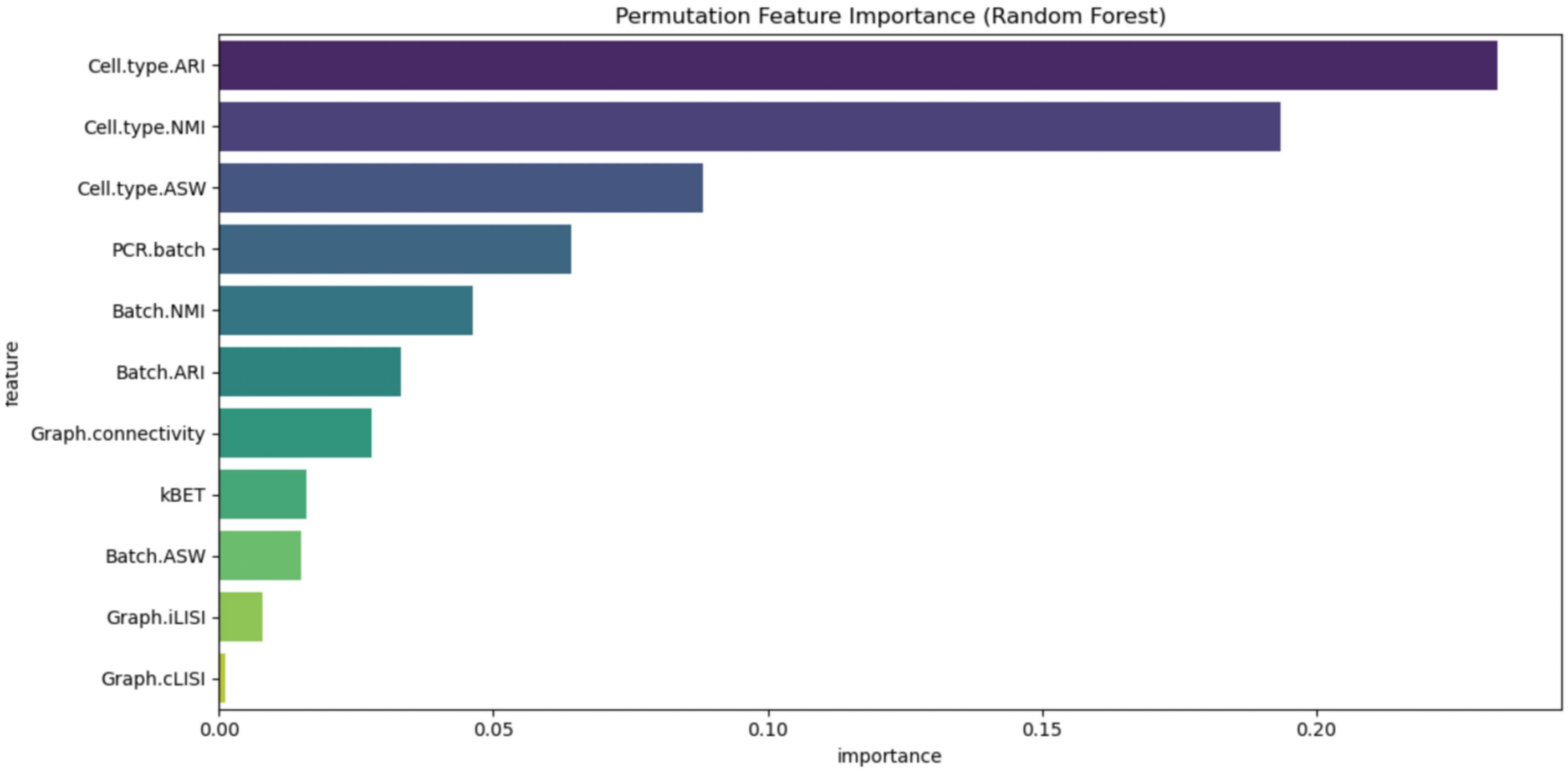

This figure is located in the construction and application of the machine learning evaluation model subsection of the results section.

Figure S3: Random forest permutation feature importance horizontal bar plot for single-cell integration evaluation metrics. Cell type-related biological conservation metrics dominate the highest importance values, while batch effect correction metrics show relatively lower contribution.

Alt text: This figure displays the results of the feature importance analysis based on the Random Forest model, using permutation methods to assess the importance of various evaluation metrics in the overall scoring.

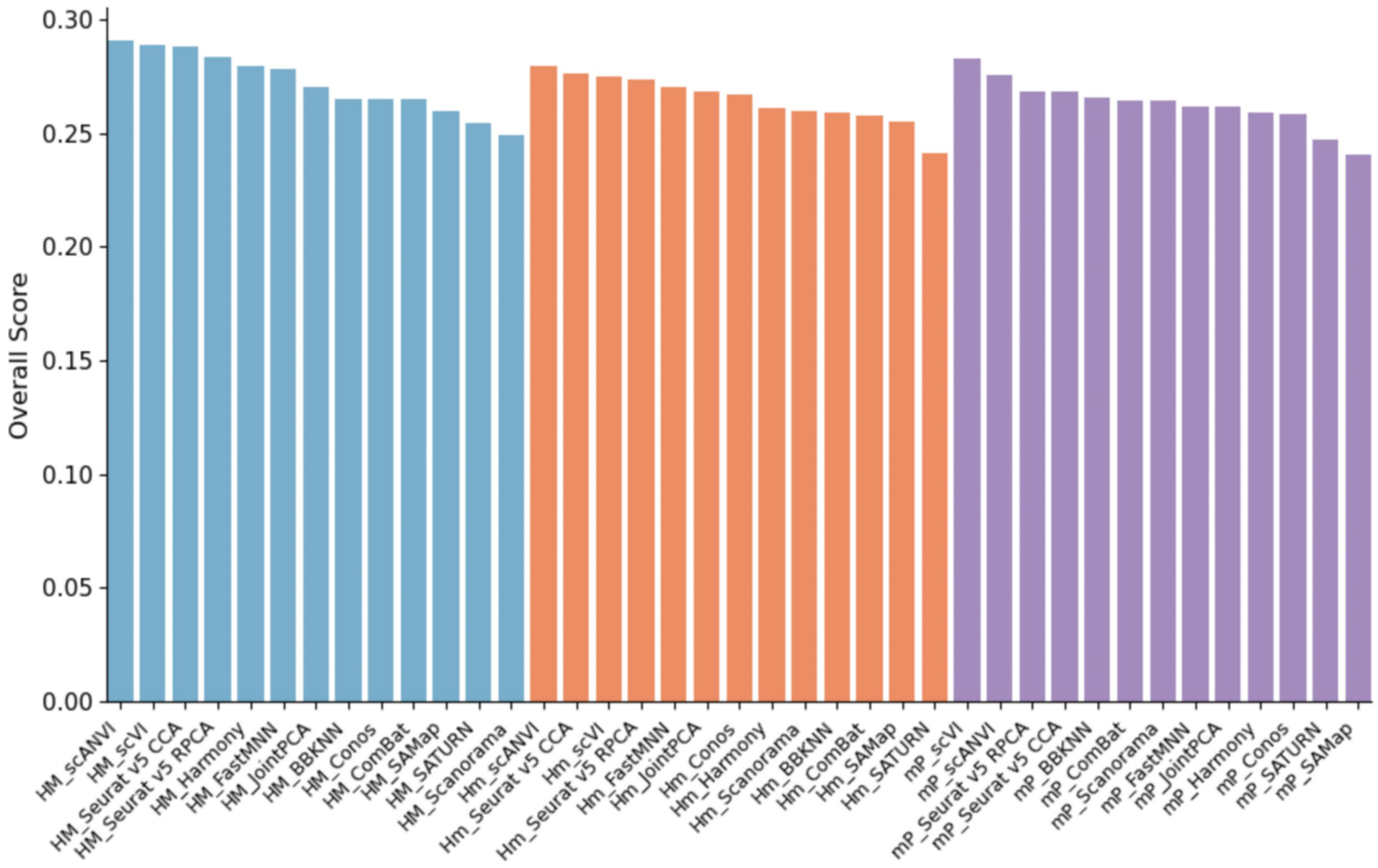

This figure is located in the systematic assessment of factors affecting cross-species data integration of the results section.

Figure S4: The overall predicted scores for integration at different evolutionary relationships. Blue represents closely related species, orange represents intermediate evolutionary species, and purple represents distantly related species. The x-axis labels each method along with its corresponding evolutionary relationships, while the y-axis denotes the scores for different integration methods. The taller the column, the higher the score.

Alt text： Bar chart showing overall performance scores of 13 integration methods across three cross-species evolutionary distance conditions.

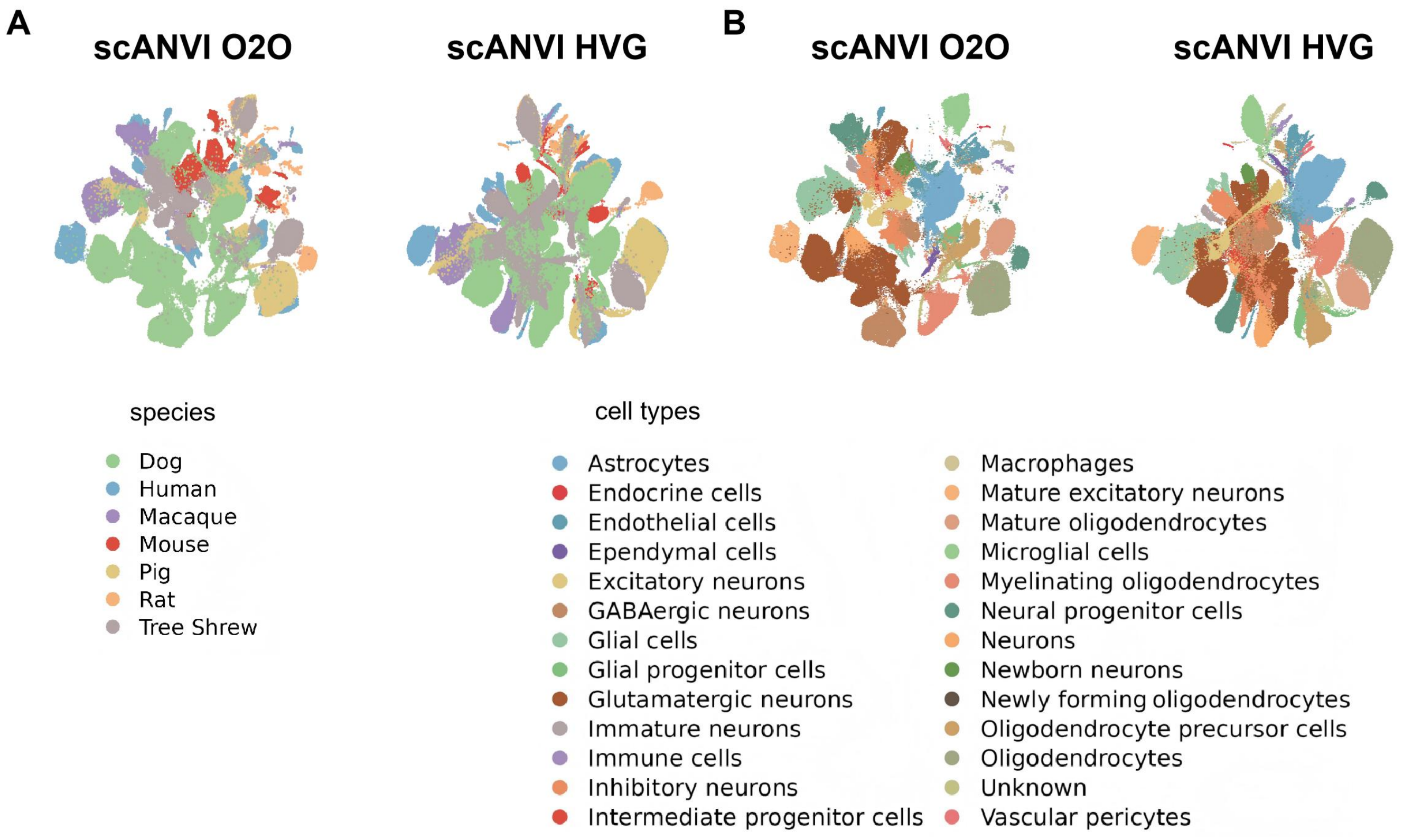

This figure is located in the systematic assessment of factors affecting cross-species data integration of the results section.

Figure S5: UMAP plots of integration tasks for 7 species, colored by species labels and cell type labels. (A) UMAP plots of species labels from scANVI integration with O2O and HVG. (B) UMAP plots of cell type labels from scANVI integration with O2O and HVG.

Alt text: UMAP plots comparing scANVI cross-species integration performance with O2O and HV gene selection. Cells are colored by species identity and annotated cell type, illustrating batch alignment and biological cluster preservation.

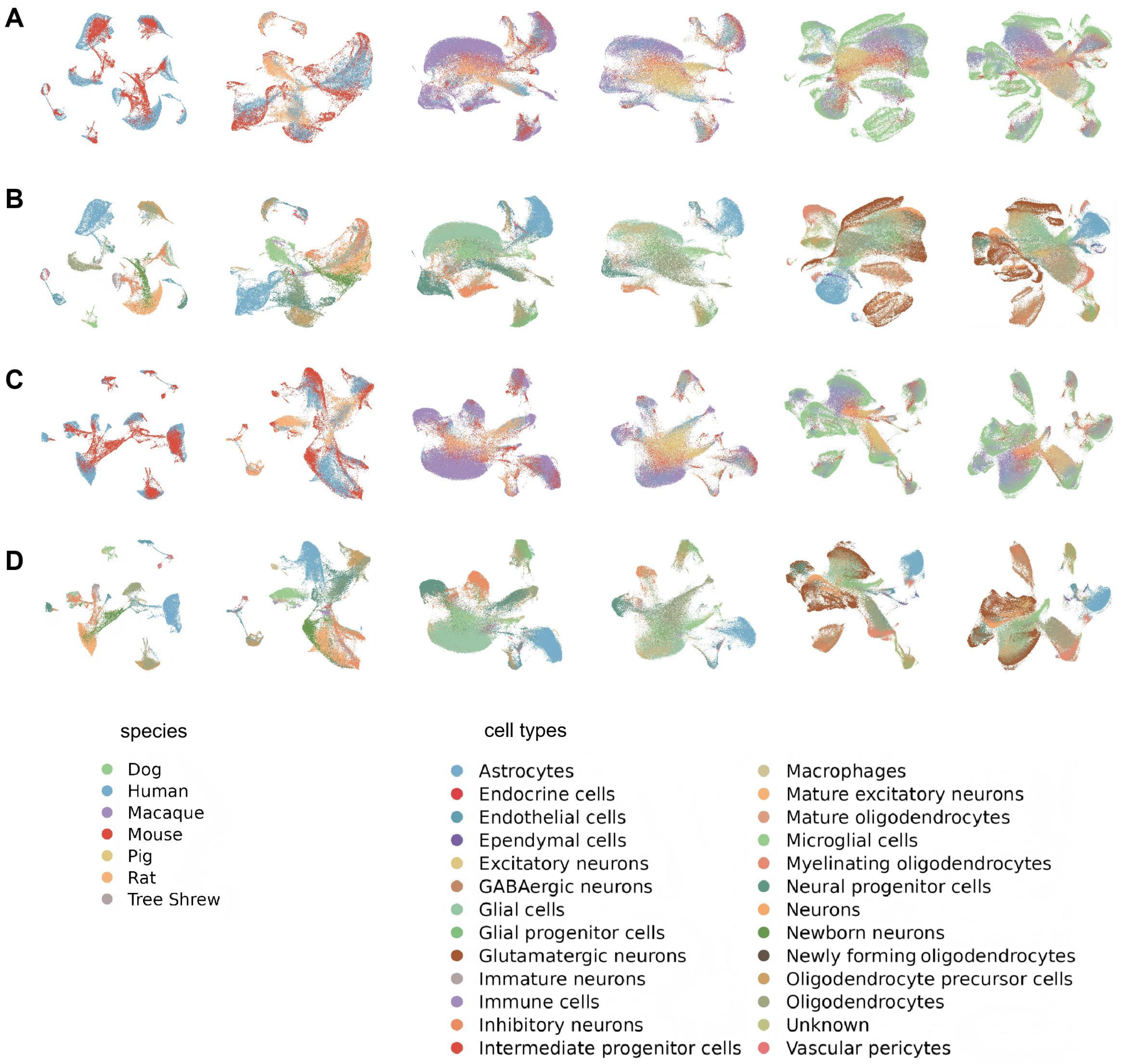

This figure is located in the systematic assessment of factors affecting cross-species data integration of the results section.

Figure S6: UMAP plots of integration tasks for seurat v5 CCA, colored by species labels and cell type labels. (A) UMAP plots of species labels from seurat v5 CCA integration with O2O. (B) UMAP plots of cell type labels from seurat v5 CCA integration with O2O. (C) UMAP plots of species labels from seurat v5 CCA integration with HVG. (D) UMAP plots of cell type labels from seurat v5 CCA integration with HVG.

Alt text： UMAP plots comparing cross-species single-cell integration results across algorithms. Cells are colored by annotated cell types, showing batch alignment and preservation of neuronal and glial cluster structures across seven species.

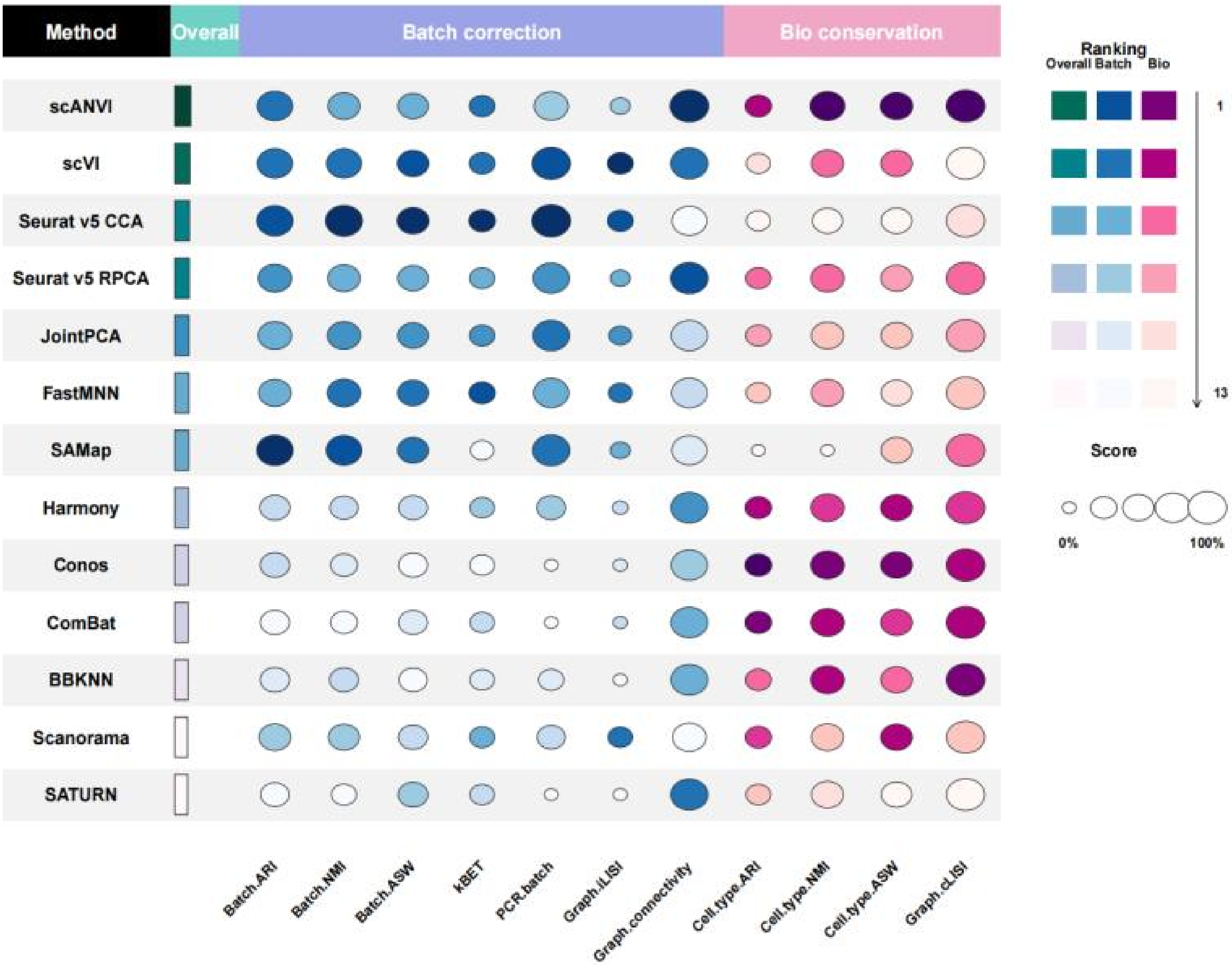

This figure is located in the development of a cross-species integration assessment website platform section.

Figure S7: The average overall scores and rankings of all methods in the PBMC cross-species integration task. This is a rating heatmap where methods with higher overall scores are ranked higher, and a darker color indicates a higher overall score. For detailed metrics, a higher score also corresponds to a darker color. The size of the circle represents the relative magnitude of the score for each specific metric, with a larger circle indicating a relatively higher score for that metric within the corresponding method.

Alt text： A comparative plot showing the ranking changes of 13 integration methods in the PBMC dataset. Left: ranks from traditional scoring formula; right: ranks from machine learning evaluation model. Connecting lines visualize individual method rank shifts and overall ranking rearrangement.

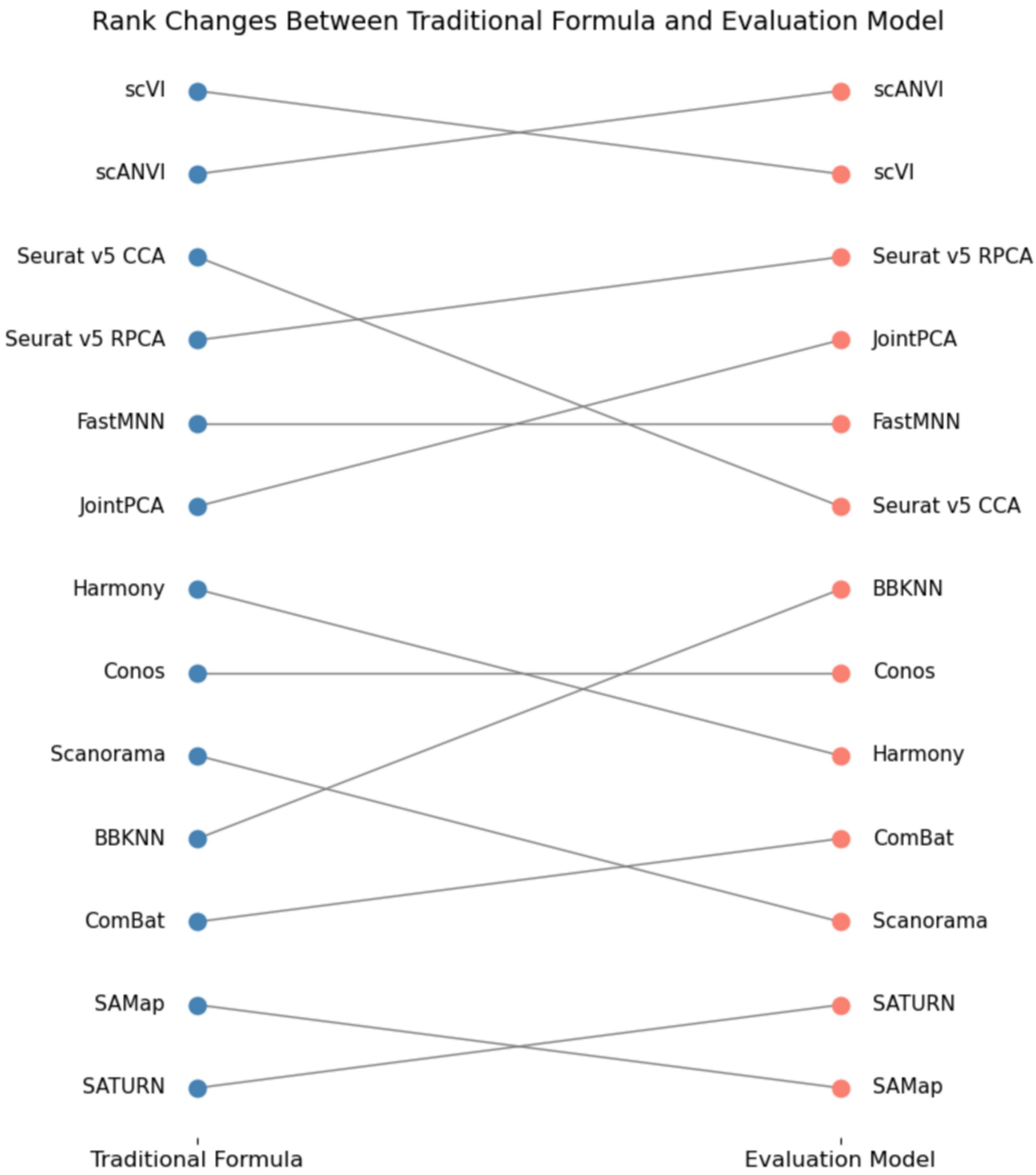

This figure is located in the discussion section.

Figure S8: Rank Changes Between Traditional Formula and Evaluation Model.This rank swap diagram illustrates performance ranking reordering of 13 cross-species single-cell integration methods between two scoring systems. The left column (blue dots) shows rankings calculated using the Traditional Formula scoring approach. The right column (red dots) shows updated rankings generated by the proposed machine learning Evaluation Model. Gray connecting lines track how each method's relative rank shifts between the two frameworks.

Alt text： Rank comparison diagram showing position changes of 13 integration methods. Left: ranks from traditional scoring formula; right: ranks from machine learning evaluation model. Connecting lines visualize individual method rank shifts and overall ranking rearrangement.

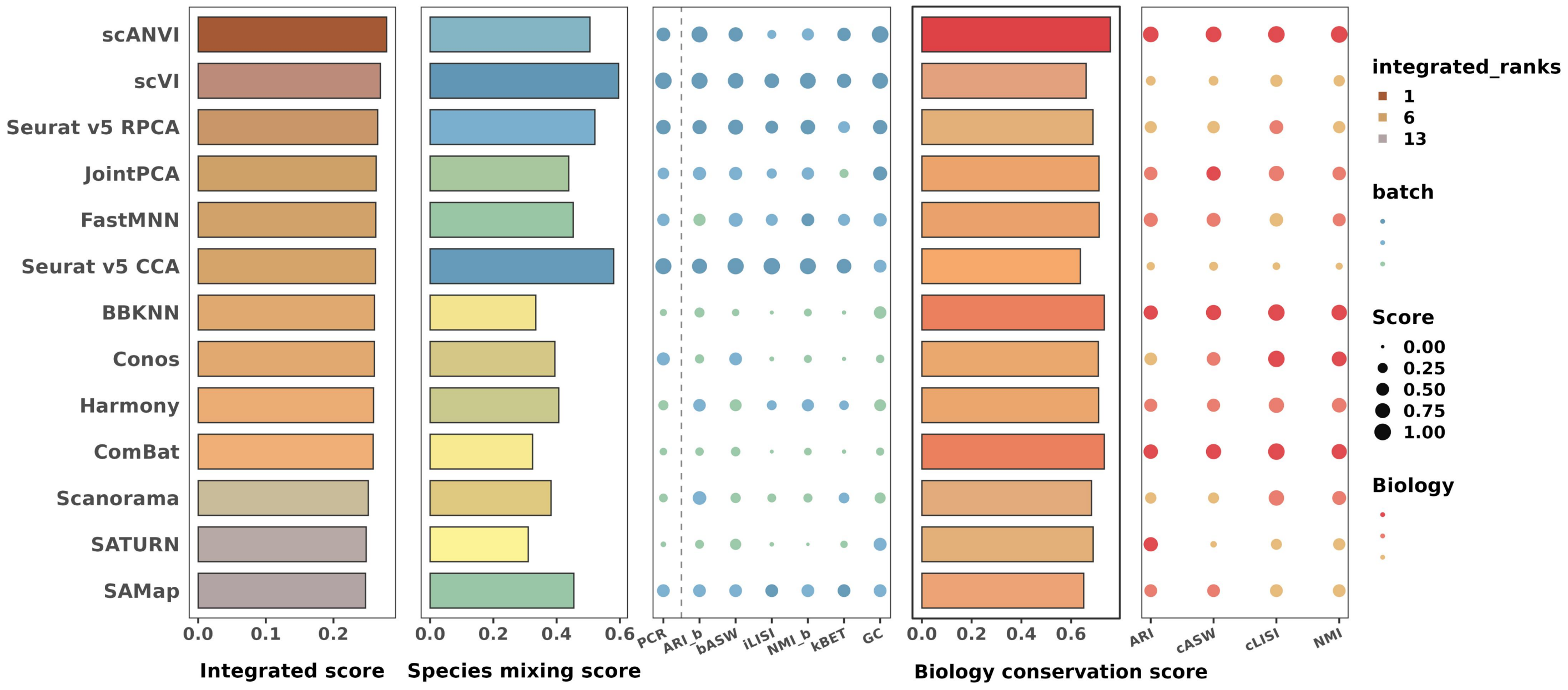

This figure is located in the discussion section.

Figure S9: Performance comparison of 13 integration methods in the cross-species integration task. From left to right, the bar plots show the integrated score, species mixing score, and biology conservation score for each method, with higher values indicating better performance. The dot plots summarize the individual metrics contributing to batch-related and biology-related evaluation, with dot size representing the normalized score and color indicating the integrated rank. Grey dash lines indicate a distinction between metrics that do not rely on cell type annotation (PCR) with metrics relying on cell type annotation.

Alt text: A multi-panel plot comparing 13 single-cell integration methods. Horizontal bar charts rank methods by integrated score, species mixing, and biology conservation. Dot plots detail the underlying batch and biology metrics, with dot size indicating score and color indicating rank. A dashed line distinguishes label-dependent from label-independent metrics. scANVI and scVI rank highest overall.

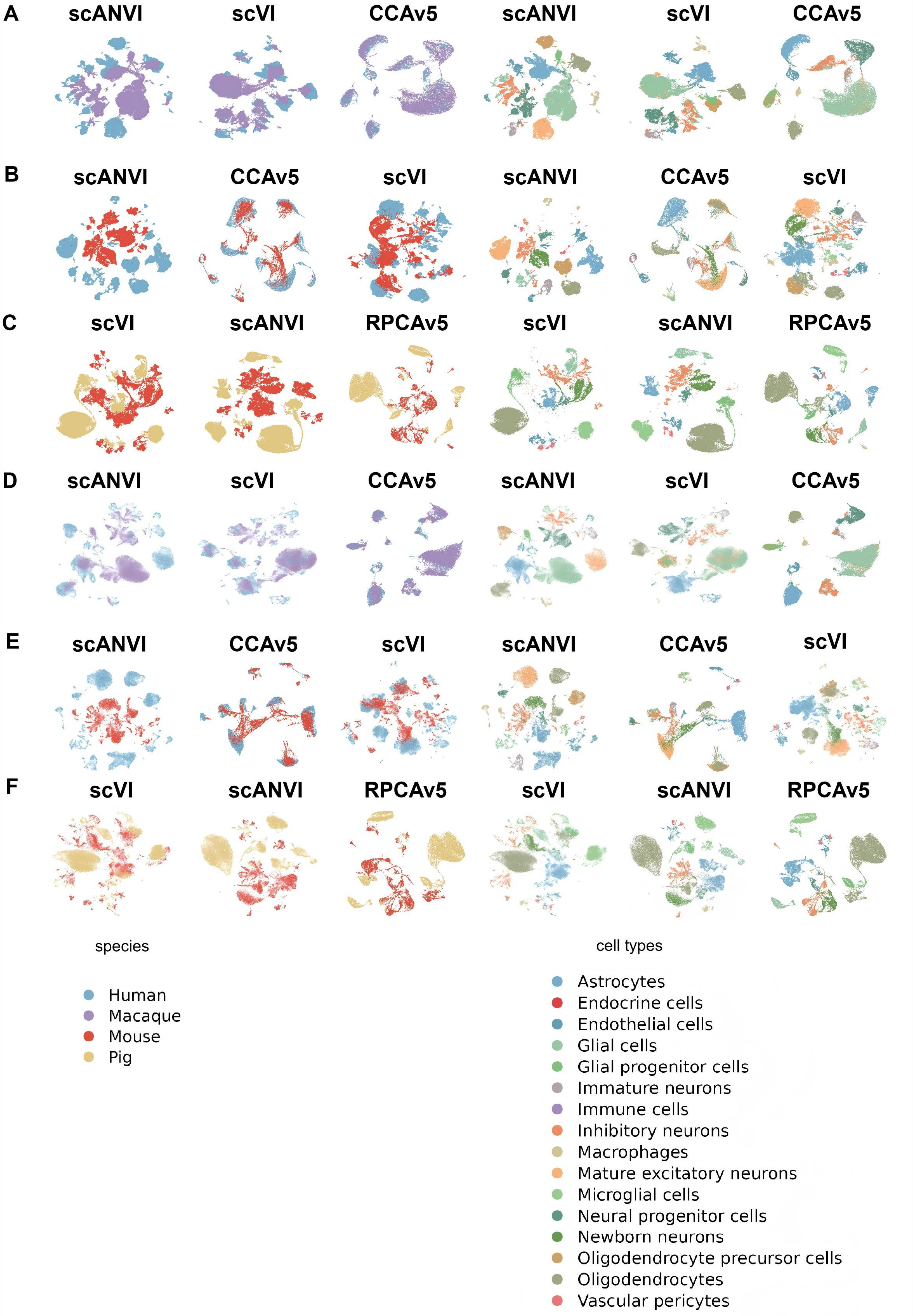

This figure is located in the discussion section.

Figure S10: UMAP plots of integration tasks for different evolutionary relationships , colored by species labels and cell type labels. (A) UMAP plots of Human and Macaque with O2O. (B) UMAP plots of Human and Mouse with O2O. (C) UMAP plots of Mouse and Pig with O2O. (D) UMAP plots of Human and Macaque with HVG. (E) UMAP plots of Human and Mouse with HVG. (F) UMAP plots of Mouse and Pig with HVG.

Alt text： Six-row multi-panel UMAP comparison of scANVI, scVI, CCAv5 and RPCA v5 cross-species integration. Cells are colored by species and annotated cell type, showing batch correction efficacy and biological cluster preservation across methods.

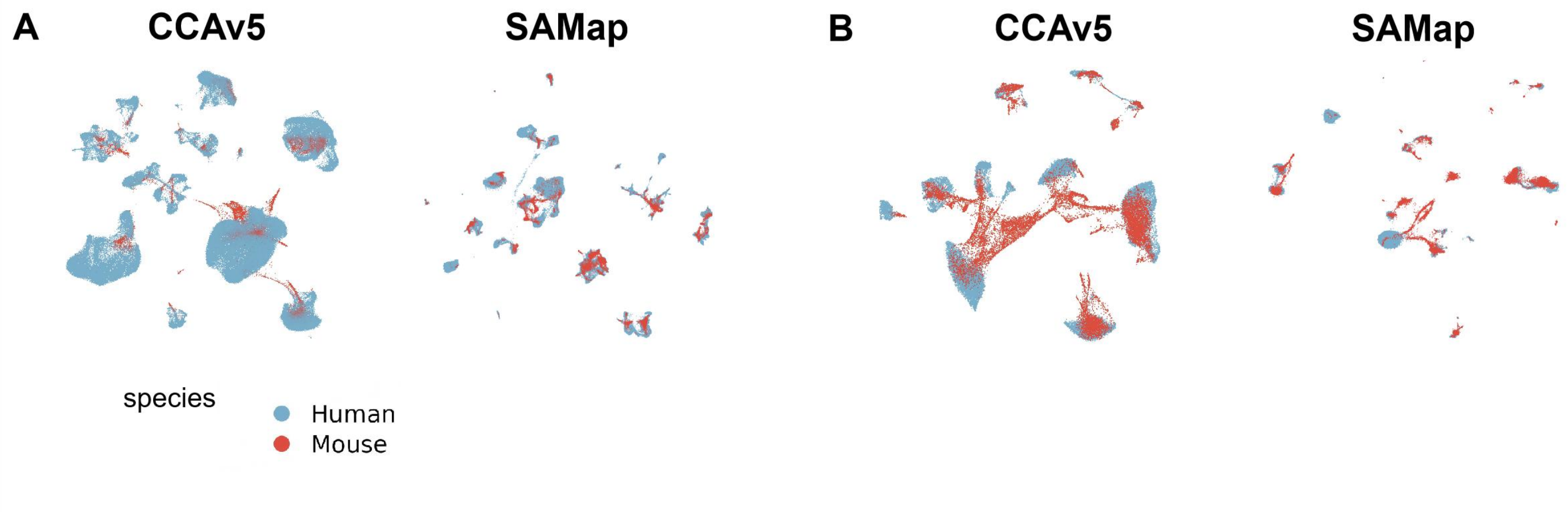

This figure is located in the discussion section.

Figure S11: Integration of imbalanced data by different methods. (A) UMAP plots from Seurat v5 CCA and SAMap when mouse cell numbers are three times that of human. (B) UMAP plots from Seurat v5 CCA and SAMap, with equivalent mouse and human cell numbers.

Alt text： UMAP comparison of CCAv5 and SAMap cross-species integration performance on imbalanced human-mouse datasets. Cells are colored by species origin, to visually demonstrate and contrast the batch correction and cell cluster alignment capabilities of the two algorithms.

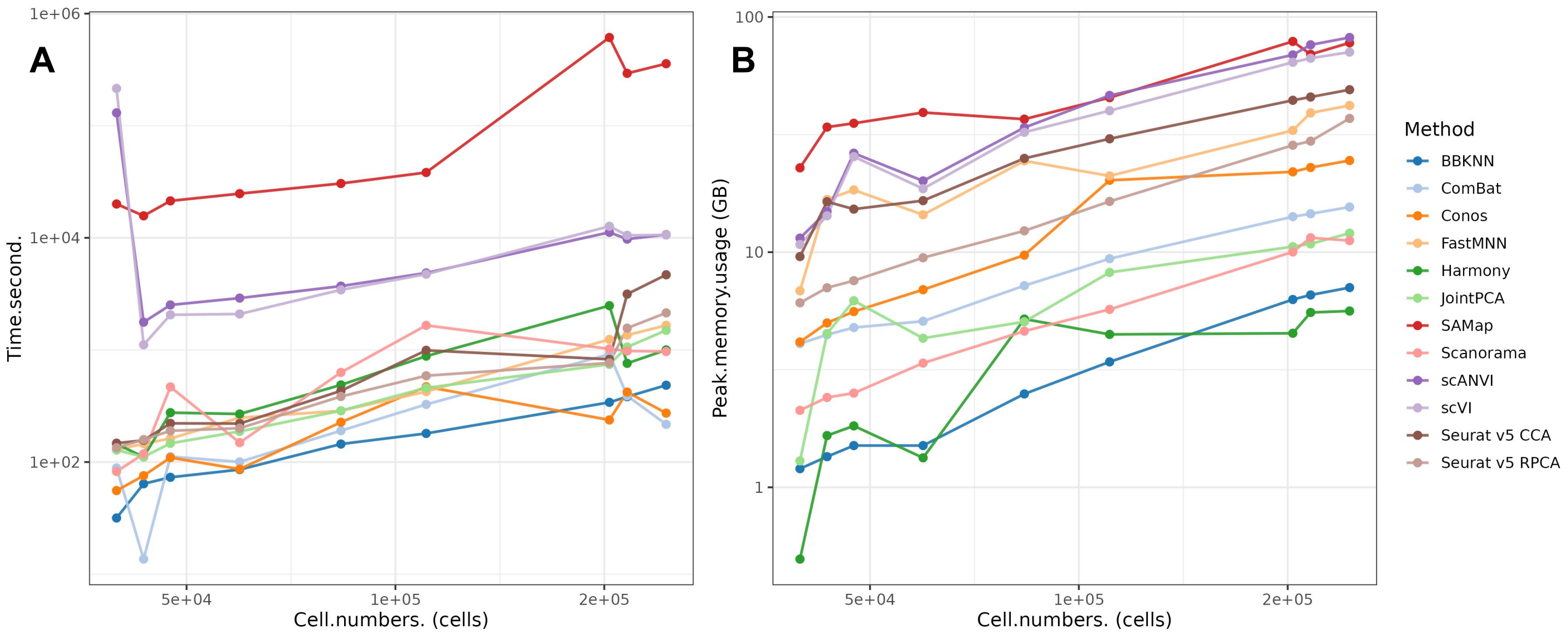

This figure is located in the discussion section.

Figure S12: Benchmark of runtime and peak memory usage for different integration methods. (A) Comparison of runtime across integration methods at different cell numbers. The x-axis represents the number of cells, and the y-axis represents runtime in seconds. (B) Comparison of peak memory usage across integration methods at different cell numbers. The x-axis represents the number of cells, and the y-axis represents peak memory usage in GB.

Alt text: A two-panel line chart. Panel A shows runtime across integration methods at different cell numbers, and Panel B shows peak memory usage across methods at different cell numbers. Each colored line represents one method.
